# Dual regulation of Arabidopsis AGO2 by arginine methylation

**DOI:** 10.1038/s41467-019-08787-w

**Published:** 2019-02-19

**Authors:** Po Hu, Hongwei Zhao, Pei Zhu, Yongsheng Xiao, Weili Miao, Yinsheng Wang, Hailing Jin

**Affiliations:** 1 Department of Microbiology & Plant Pathology, Center for Plant Cell Biology, Institute for Integrative Genome Biology, University of California, 900 University Avenue, Riverside, CA 92521 USA; 2 Department of Chemistry, Center for Plant Cell Biology, Institute for Integrative Genome Biology, University of California, 900 University Avenue, Riverside, CA 92521 USA; 3 Present Address: Department of Plant Protection, Nanjing Agriculture University, Nanjing, 210095 China

## Abstract

Argonaute (AGO) proteins are core components of RNA interference (RNAi) but the mechanisms of their regulation, especially at the post-translational level, remain unclear. Among the ten AGOs in Arabidopsis, only AGO2 is induced by bacterial infection and is known to positively regulate immunity. Here we show that the N-terminal domain of AGO2 is enriched with arginine-glycine RG/GR repeats, which are methylated by protein arginine methyltransferase5 (PRMT5). Arginine methylation has dual functions in AGO2 regulation. Methylated arginine residues can promote AGO2 protein degradation and are also bound by Tudor-domain proteins (TSNs), which can degrade AGO2-associated small RNAs (sRNAs). PRMT5 is down-regulated during infection and the *prmt5* mutant is more resistant to bacteria. We speculate that reduced PRMT5 expression during infection may lead to reduced arginine methylation of AGO2, resulting in accumulation of both AGO2 and, via reduced interaction with TSNs, accumulation of AGO2-associated sRNAs, to promote plant immunity. These results reveal that both the arginine methylation writer (PRMT5) and readers (TSNs) can regulate AGO2-mediated RNAi.

## Abstract (web summary)

AGO2 is a core component of the RNAi machinery and contributes to plant immunity during bacterial infection. Here the authors show that AGO2 activity is suppressed by arginine methylation which not only promotes AGO2 degradation but also recruits TSN proteins to degrade AGO2-associated small RNAs.

## Introduction

Microbial pathogens cause detrimental and deadly human and animal diseases, as well as severe yield losses in crop plants^1–3^. RNA interference (RNAi) mediated by small RNAs (sRNAs) plays a critical role in the immune responses of both animals and plants^4–7^. Different sRNAs are sorted into distinct AGO proteins to direct silencing of target genes with complementary sequences by mRNA degradation, translational inhibition, or chromatin modification^8,9^. Dysfunction of AGO proteins has been linked to many animal and plant diseases^10–12^. For example, human AGO2 is involved in tumorigenesis and is often overexpressed in various cancers^13^. Human AGO2 and the associated microRNAs (miRNAs) regulate gene expression in all major types of immune cells and contribute to innate and adaptive immunity^14,15^. T-cell activation induces ubiquitylation and degradation of AGO2, resulting in rapid remodeling of the miRNA repertoire and target gene expression^16^. Similarly, plant AGOs also play an important role in host immune responses against pathogen infection^5,17,18^. Among the 10 *Arabidopsis* AGOs, only AGO2 is highly induced by bacterial infection and positively regulates antibacterial defense responses^19^. The *Arabidopsis ago2* mutant is more susceptible to both virulent and avirulent strains of *Pseudomonas syringae*. One of the most abundant sRNAs that is associated with AGO2 is miR393*, which suppresses a SNARE protein gene to promote secretion of antimicrobial peptides and inhibit bacterial growth^19^. The other strand of this miRNA duplex miR393 is loaded into another AGO protein, Arabidopsis AGO1, and silences auxin receptors to shift energy from plant growth to innate immunity^20^. The *ago1* mutant displays attenuated plant immunity triggered by pathogen-associated molecular patterns^21^.

Post-translational modifications (PTMs) of proteins can largely affect protein function by either suppressing or enhancing protein activities, or, by switching between two different functions^22,23^. Many proteins that catalyze the addition of PTMs (writers), remove these PTMs (erasers), or bind specific PTMs to transduce downstream signals (readers) have been identified^24,25^. Dysregulation or mutation in protein modification enzymes is often associated with cancers and other human diseases^26–31^. Previous studies on PTMs of AGO proteins have focused mainly on AGO phosphorylation, which has been linked to stress responses and diverse pathological processes, including cancer^32^. So far, little is known about any role of AGO PTMs in regulating host immunity.

Here, we show that the *N*-terminal of *Arabidopsis* AGO2 is subjected to symmetric arginine dimethylation by the arginine methyltransferase PRMT5. We demonstrate that arginine methylation can regulate both AGO2 protein stability and AGO2 interaction with Tudor-domain proteins (TSNs) that can regulate accumulation of AGO2-associated sRNAs.

## Results

### PRMT5 interacts with Arabidopsis AGO2 in vivo

**Fig. 1:**
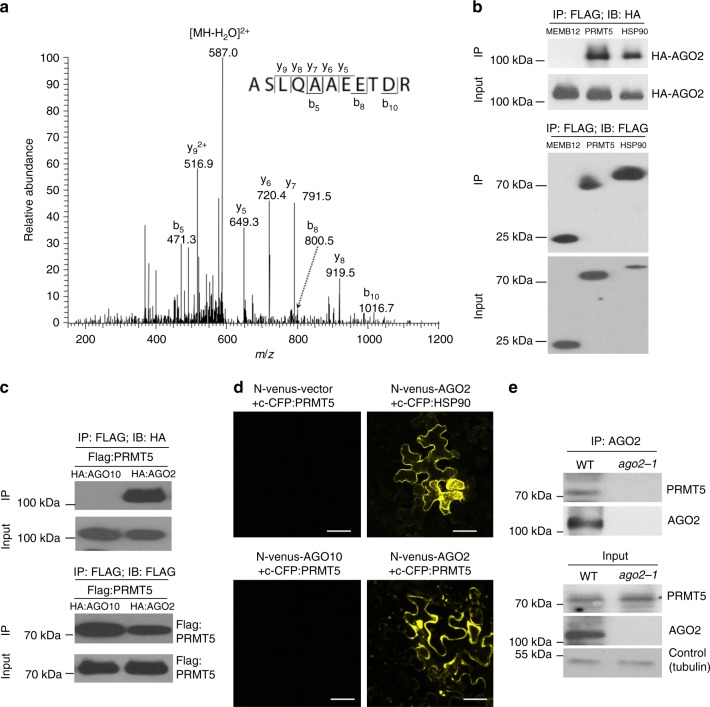
PRMT5 interacts with AGO2. **a** Representative MS/MS of the [M + 2 H]^2+^ ion of a PRMT peptide identified from co-immunoprecipitated (IP) complexes associated with HA-tagged AGO2 (HA:AGO2). Displayed in the inset is a scheme summarizing the observed fragment ions for the peptide. **b** Association of *Arabidopsis* PRMT5 and AGO2 was detected in a co-IP assay when transiently expressed in *Nicotiana benthamiana*. HA:AGO2 was transiently expressed along with Flag:PRMT5, Flag:HSP90 (positive interaction control), or Flag:MEMB12 (negative interaction control) in *Nicotiana benthamiana*. Protein complexes were immunoprecipitated using anti-Flag antibody to pull down PRMT5 and probed with antibodies against Flag or HA tags. **c** Flag:PRMT5 interacts only with HA:AGO2 but not with HA:AGO10. Protein complexes were immunoprecipitated using anti-Flag antibody and probed with antibodies against Flag or HA tags after being transiently expressed in *N. benthamiana*. **d** Bimolecular fluorescence complementation assay in *N. benthamiana* leaf epidermal cells shows that N-venus:AGO2 interacts with c-CFP:PRMT5. Yellow fluorescence is not reconstituted in the absence of an interacting bait protein (negative control, left upper panel) or using N-venus:AGO10 as a bait protein (negative control, left bottom panel), while fluorescence is observed when co-expression of N-venus:AGO2 with c-CFP:HSP90 (positive control, right upper panel) and c-CFP:PRMT5. The scale bar represents 50 µm. **e** Association of PRMT5 and AGO2 was observed in vivo in the wild-type (WT) *Arabidopsis thaliana* plants (Col-0), but not in the *ago2-1* mutant by the co-IP assay. Native antibodies to AGO2 and PRMT5 were used to pull down AGO2 and detect PRMT5, respectively. Tubulin was used to show equal protein loading

We have demonstrated that *Arabidopsis* AGO2 is strongly induced upon infection by *Pseudomonas syringae* pv tomato (*Pst*) carrying an effector protein, avrRpt2, and positively regulates plant antibacterial immunity^19^. To investigate the regulatory mechanism of AGO2, we isolated AGO2 interacting proteins by co-immunoprecipitation (co-IP) coupled with mass spectroscopy (MS) analysis in *Arabidopsis*, as described in human AGO studies^33^. The transgenic complementary plants expressing tagged AGO2 protein under its native promoter *pAGO2:3HA:AGO2* in the *ago2-1* mutant background were used for the analysis after infection by *Pst* (*avrRpt2*)^34^. The transgenic plants expressing the reporter gene *β-glucuronidase* (p*AGO7:gus*) were used as a negative control. We identified 70 AGO2-associated proteins that were only associated with *pAGO2:3HA:AGO2* but not with p*AGO7:gus*, by using the label-free quantification (LFQ) intensity value (Supplementary Data 1). As expected, HSP90 and HSP70, two heat-shock proteins previously reported to be associated with animal AGO2 RISC^35,36^, were found to be associated with *Arabidopsis* AGO2. PRMT5, a type II protein arginine methyltransferase, was also identified (Fig. 1a and Supplementary Fig. 1a). PRMT5 catalyzes the formation of monomethylarginine (MMA) and symmetric dimethylarginine (sDMA), which is involved in regulating many animal developmental and pathological processes^28,37^. However, the function of PRMT5 in regulating AGO proteins and RNAi, especially in host immune responses against pathogen infections, has not been previously reported in any system.

The interaction of *Arabidopsis* PRMT5 and AGO2 was confirmed by co-IP and by bimolecular fluorescence complementation (BiFC) when transiently expressed in *Nicotiana benthamiana* (Fig. 1b, d). PRMT5 was associated only with *Arabidopsis* AGO2 but not with AGO10 (Fig. 1c, d). This interaction was further affirmed in wild-type *Arabidopsis* by co-IP using native antibodies that recognize AGO2 and PRMT5 proteins (Fig. 1e and Supplementary Fig. 1b). As a control, PRMT5 was not detected in the *ago2-1* mutant (Fig. 1e). Taken together, these results demonstrate that PRMT5 is associated with AGO2 in vivo.

### PRMT5 directs symmetric arginine dimethylation of AGO2

**Fig. 2:**
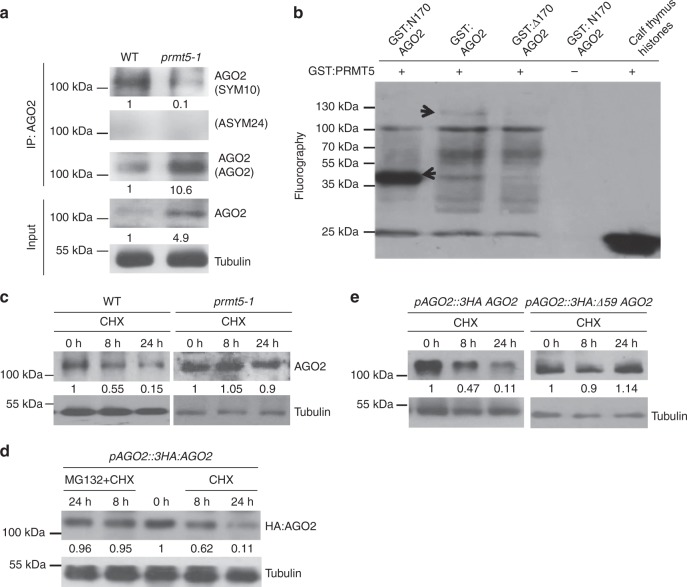
PRMT5 catalyzes AGO2 methylation at the AGO2 N-terminal GAR region and regulates AGO2 degradation. **a** In vivo methylation of AGO2 requires PRMT5. AGO2 was immunoprecipitated with the anti-AGO2 antibody and symmetrically dimethylated arginines (sDMA) were detected using the anti-symmetric-dimethyl-arginine antibody (SYM10). The anti-asymmetric-dimethyl-arginine antibody (ASYM24) was used to detect aDMA. **b** The *N*-terminal region of AGO2 is required for PRMT5-mediated methylation of AGO2 in vitro. Recombinant, GST-tagged AGO2 proteins were incubated with GST:PRMT5 in the presence of the methyl donor [^3^H] *S*-adenosyl-L-methionine. The 170 residue *N*-terminal region of AGO2 (GST:N170 AGO2) and full-length AGO2 protein (GST:AGO2) are both methylated in the presence of GST:PRMT5 (arrows), but AGO2 proteins with N-terminal truncations (GST:∆170 AGO2) are not. **c** AGO2 degradation depends on the presence of PRMT5. Protein synthesis was inhibited through 50 µM cycloheximide (CHX) treatment to show that AGO2 levels decrease over time in WT plants, while AGO2 levels are maintained in the *prmt5–1* mutant. **d** AGO2 degradation is 26S proteasome-dependent. Treatment with the 26S proteasome inhibitor MG132 (40 µM) prevents AGO2 degradation in the *ago2-1* complementary plants (*pAGO2:3HA:AGO2*). **e** Levels of AGO2 were compared in *ago2-1* expressing full-length AGO2 (*pAGO2:3HA:AGO2)* or truncated AGO2 (*pAGO2:3HA:∆59 AGO2)* in the presence of 50 µM CHX over 0, 8, and 24 h. The protein levels of full-length AGO2 and truncated AGO2 (*∆59*) were tested using anti-HA antibodies. Tubulin levels were used to show equal loading for all western blots. In all panels, signal intensity was calculated using ImageJ

It is notable that arginine–glycine/glycine-arginine (RG/GR) repeats, defined as the glycine–arginine-rich (GAR) domain, are highly enriched in the *N*-terminus of *Arabidopsis* AGO2 and AGO3, modestly so in AGO1, and not at all in other Arabidopsis AGOs or in animal AGO2 (Supplementary Figs. 2, 3a). To determine whether PRMT5 methylates arginine residues of AGO2, we examined the status of arginine methylation of AGO2. The guanidine group of arginine can potentially be methylated into three different ways: monomethylation, symmetrical dimethylation, and asymmetrical dimethylation. We used the antibodies anti-SYM10 and anti-ASYM24 that specifically recognize sDMA and asymmetric dimethylarginine (aDMA) modifications, respectively, to examine the methylation status of AGO2. A strong sDMA signal, but not an aDMA signal, was detected in AGO2 protein purified from wild-type Col-0 plants, indicating that AGO2 has mostly sDMA modifications (Fig. 2a). The symmetric dimethylation of arginine is PRMT5-dependent as revealed by the finding that the sDMA signal was almost eliminated in the *prmt5–1* T-DNA knockout mutant (Fig. 2a). To further confirm that arginine methylation of AGO2 is mediated by PRMT5, we performed an in vitro reconstitution assay by co-incubation of the recombinant full-length GST:AGO2 and GST:PRMT5 in the presence of the [^3^H]-labeled methyl group donor [methyl-^3^H] *S*-adenosyl-L-methionine. Full-length GST:AGO2 protein was clearly methylated (Fig. 2b and Supplementary Fig. 4).

We hypothesize that PRMT5 binds with and mediates methylation at the *N*-terminal GAR domain (170 aa fragment) of AGO2 (Supplementary Fig. 3a). Indeed, recombinant HIS-tagged PRMT5 was pulled down by the *N*-terminal 170 aa fragment of AGO2 (GST:N170 AGO2) as well as full-length GST:AGO2, but not by the truncated AGO2 lacking the first 170 residues (GST:Δ170 AGO2) (Supplementary Fig. 3b). As expected, a strong sDMA signal was detected on GST:N170 AGO2, but not on the truncated GST:Δ170 AGO2 (Fig. 2b). These results confirmed that PRMT5 directs symmetric arginine dimethylation of AGO2 at its *N*-terminal GAR domain.

### Arginine methylation leads to AGO2 degradation

Interestingly, although the level of methylated AGO2 was largely reduced in the *prmt5–1* mutant in comparison with the wild-type plants, more AGO2 proteins were accumulated in the total protein extract as well as the AGO2-IP fraction in *prmt5-1* (Fig. 2a). This result led us to hypothesize that arginine methylation may promote AGO2 degradation. To monitor the degradation of AGO2, we first treated the wild-type (WT) plant and the *prmt5-1* mutant seedlings with cycloheximide (CHX), which blocks mRNA translation. AGO2 protein levels were reduced in WT plants over time, but remained high in the *prmt5-1* mutant in which arginine methylation was mostly abolished (Fig. 2c). Similar results were observed in the transgenic plants, *pAGO2:3HA:AGO2*, treated with CHX (Fig. 2d). Furthermore, AGO2 degradation was inhibited by MG132, a 26S proteasome inhibitor in *Arabidopsis* (Fig. 2d) and in the *N. benthamiana* transient co-expression assay (Supplementary Fig. 5). These results support the conclusion that PRMT5-mediated arginine methylation promotes 26S proteasome-dependent degradation of AGO2. To further confirm this result, co-expression analysis of PRMT5 with full-length AGO2 or truncated versions of AGO2 was performed in *N. benthamiana*. We found that the deletion of the first 59 aa of AGO2, which contains 13 out of the 18 RG/GR repeats, greatly stabilized AGO2 protein levels, while deletion of the next 62 aa of AGO2, which contains only three RG/GR repeats, only modestly stabilized AGO2 levels (Supplementary Figs. 3a and 6a). Furthermore, accumulation of YFP-tagged full-length AGO2, but not YFP:*Δ59AGO2*, was largely reduced when co-expressing with PRMT5, which methylated arginine in the GAR motif of AGO2 (Supplementary Fig. 6b). Similar results were obtained in vivo in the transgenic *ago2-1 Arabidopsis* mutants expressing either full-length AGO2 (*pAGO2:3HA:AGO2)* or truncated AGO2 (*pAGO2:3HA:Δ59AGO2*). The truncated AGO2 accumulated to a higher level than the full-length AGO2 (Supplementary Fig. 7), and AGO2 degradation after CHX treatment was largely abolished in the *pAGO2:3HA:Δ59AGO2/ago2* plants (Fig. 2e). These results indicate that PRMT5-mediated arginine methylation of the *N*-terminal GAR region of AGO2 leads to AGO2 degradation.

### PRMT5 is downregulated by bacterial infection

**Fig. 3:**
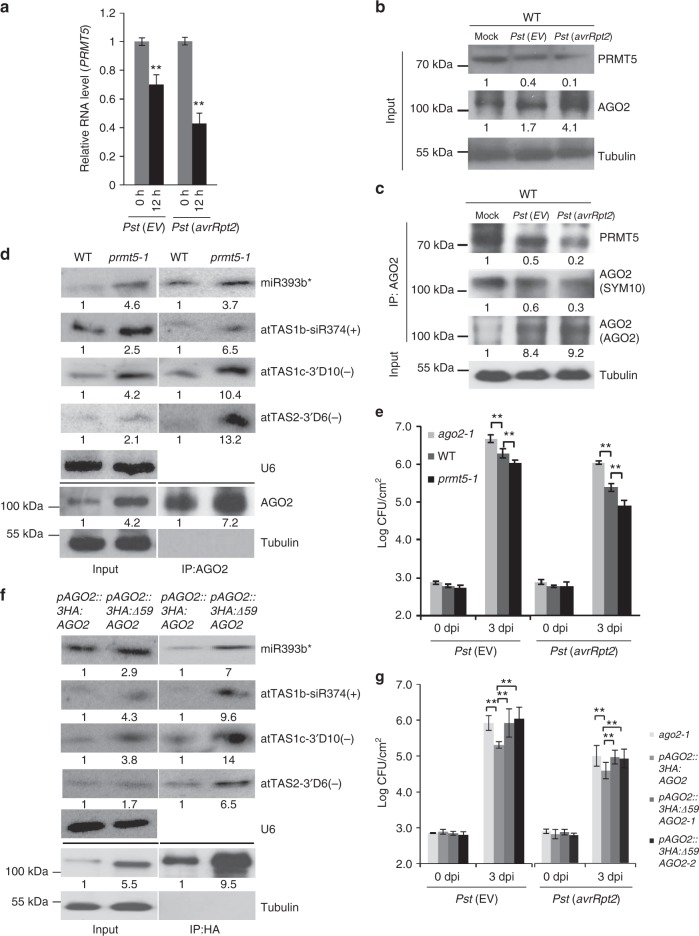
PRMT5 modulates plant innate immunity. **a** *PRMT5* expression is downregulated in leaves 12 h after bacterial infection with *Pst* (*EV*) or an avirulent strain *Pst* (*avrRpt2*). Error bars represent the standard deviation. **b** PRMT5 protein expression is downregulated in leaves 12 h after bacterial infection, whereas the level of AGO2 protein is induced. **c** The levels of AGO2 methylation and the PRMT5 protein are reduced after bacterial infection, whereas the AGO2 protein is accumulated to a higher level. Protein levels were measured in WT leaf tissue 12 hours post inoculation. **d** Accumulation of miRNA393b*, atTAS1b-siR374(+), atTAS1c-3’D10(–), and atTAS2–3’D6(–) is higher in the *prmt5–1* mutant compared to the wild type (Col-0) before and after AGO2 IP. **e** The *prmt5–1* mutant shows enhanced resistance 3 days post inoculation with *Pst* (*EV*) and *Pst* (*avrRpt2*) strains compared to *ago2-1* and WT controls. **f** The levels of AGO2 protein and the AGO2-associated sRNAs are higher in *pAGO2:3HA:∆59 AGO2* lines. **g** Two independent transgenic lines, *pAGO2:3HA:∆59 AGO2-1* and *pAGO2:3HA:∆59 AGO2-2*, were more susceptible to *Pst (avrRpt2)*, and reflects similar bacterial growth levels in the *ago2-1* mutant 3 dpi. sRNA levels were determined by Northern blot using U6 RNA to show equal loading. Tubulin levels were used to show equal loading for all Western blots. Error bars represent the standard deviation from the mean of ten 8-mm leaf disk measurements. Asterisks indicate mean values significantly different than wild-type controls using the Student’s T test (**P-value < 0.01). In all panels, signal intensity was calculated using ImageJ

We further discovered that both the RNA and protein levels of *PRMT5* were downregulated after infection with either a virulent strain of *Pst* carrying an empty vector (EV) or the avirulent strain *Pst* (*AvrRpt2*) (Fig. 3a, b). Although the AGO2 protein accumulated to a higher level after bacterial challenge, less sDMA of AGO2 was detected after infection and this decrease was correlated with the decreased level of PRMT5 (Fig. 3b, c). Consistent with this result, the level of AGO2 was higher in the *prmt5-1* mutant than wild-type plants, and increased amounts of AGO2-associated sRNAs, including miR393b*^19^ and *trans*-acting small interfering RNAs (ta-siRNAs:AtTAS1b-siR374(+) and AtTAS1c-3’D10(−); AtTAS2–3’D6(−))^34,38,39^, were detected in the AGO2-IP fraction in the *prmt5-1* mutant (Fig. 3d). Furthermore, the expression levels of the corresponding targets of these AGO2-associated sRNAs were significantly decreased in the *prmt5-1* mutant as compared with the wild type (Supplementary Fig. 8a and b). As expected, the *prmt5-1* mutant had the opposite effect of *ago2-1* and was more resistant to *Pst* infection compared with the wild-type control (Fig. 3e), indicating that PRMT5 negatively regulates both AGO2 and plant bacterial resistance.

To test whether the *pAGO2:3HA:Δ59AGO2* plants, that accumulated higher levels of AGO2, are also more resistant to *Pst* infection as observed in *prmt5–1*, we performed a bacterial infection assay and examined sRNA loading into AGO2. Although higher AGO2-associated sRNA loading was observed in *pAGO2:3HA:Δ59AGO2* transgenic plants (Fig. 3f), the mRNA targets were not silenced (Supplementary Fig. 9a and b), suggesting that the silencing function of AGO2 was impaired after the *N*-terminal deletion. The *pAGO2:3HA:Δ59AGO2* plants showed higher susceptibility to bacterial infection, similar to the *ago2-1* mutant phenotype, further suggesting that the *N*-terminal GAR domain is important for AGO2 function in addition to its role in modulating AGO2 stability (Fig. 3g).

### Arginine-methylated AGO2 is bound by Tudor-domain proteins

**Fig. 4:**
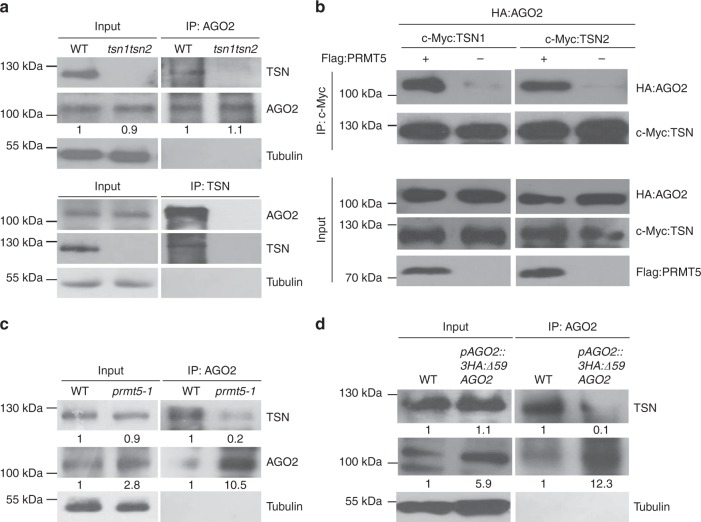
*Arabidopsis* TSN proteins bind to arginine-methylated AGO2. **a** Interaction between TSNs and AGO2 was examined in *Arabidopsis* by co-IP assays. Native antibodies of TSN and AGO2 proteins were used for pull-downs and western-blot analysis. **b** PRMT5-directed arginine methylation mediates the interaction between AGO2 and TSN1 and TSN2 assayed by co-IP analysis. **c** TSNs interact with AGO2 in a PRMT5-dependent manner in *Arabidopsis*. **d** Arginine methylation in the AGO2 *N*-terminal region is essential for interactions with TSN proteins. Deletion of the *N*-terminal region largely reduced the binding with TSNs. Tubulin levels were used to show equal loading for all Western blots. In all panels, signal intensity was calculated using ImageJ

In animals, the *N*-terminal domain of a germline-specific subgroup of AGOs, PIWI proteins, contains 2–7 RG repeats^40–42^, which are also methylated by PRMTs. The sDMAs of PIWI proteins are recognized and bound by Tudor proteins, which regulate silencing and gametogenesis^40,42–46^. Although plants lack PIWI subfamily proteins, they do have Tudor-like proteins. We hypothesized that plant Tudor-like proteins may also serve as sDMA readers of AGO2. In *Arabidopsis*, we identified two Tudor-like proteins, TSN1 and TSN2, which contain four staphylococcal/micrococcal-like nuclease (SN) domains and a Tudor domain that are homologous to human Tudor protein SND1 (Supplementary Fig. 10). Interaction between AGO2 and TSN was detected in vivo in *Arabidopsis* using reciprocal co-IP assays with native antibodies that recognize AGO2 or TSN proteins (Fig. 4a). Indeed, AGO2 interacts with both TSN1 and TSN2 in the presence of PRMT5 when co-expressed in *N. benthamiana* but AtAGO10, which lacks the *N*-terminal GAR domain, does not (Supplementary Fig. 11a). In vitro co-IP assays using recombinant proteins further confirmed that the interaction between AGO2 and TSNs is PRMT5-dependent, and deletion of the entire GAR domain of AGO2 completely abolished AGO2 and TSN protein binding (Supplementary Fig. 11b). Similar results were obtained in the transient co-expression assays in *N. benthamiana* (Fig. 4b and Supplementary Fig. 11c). Although the total amount of TSN protein was not altered in the *prmt5-1* mutant, the amount of TSN protein that was pulled down with AGO2 was almost completely abolished in the *prmt5-1* mutant (Fig. 4c), confirming that the interaction between AGO2 and TSN proteins is dependent on PRMT5-mediated arginine methylation. Partial deletion of the *N*-terminal GAR domain largely suppressed the binding between AGO2 and TSN proteins, even though the protein level of Δ59AGO2 was elevated (Fig. 4d).

### AGO2-bound TSNs degrade AGO2-associated sRNAs

**Fig. 5:**
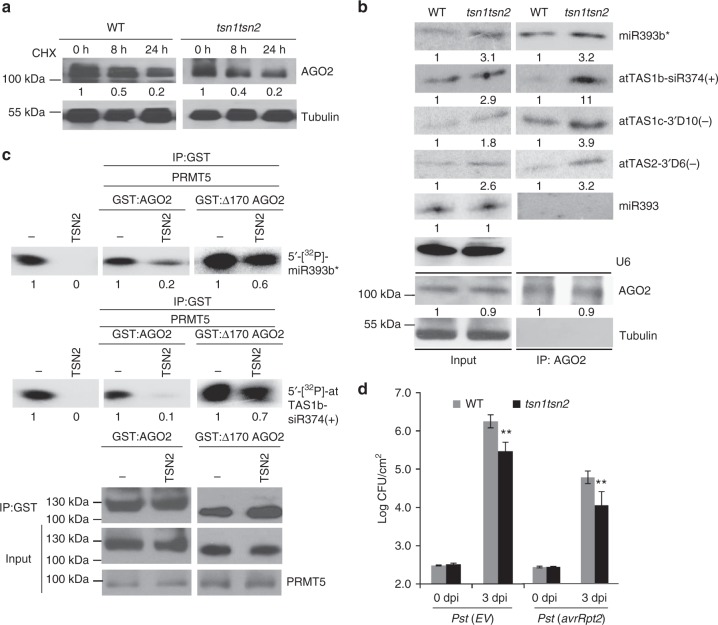
TSN mediates sRNA degradation, which can be inhibited by deletion of the GAR domain of AGO2. **a** TSN proteins do not affect AGO2 degradation. The double-knockout mutant *tsn1/tsn2* shows that AGO2 degradation is comparable with the WT control. **b** Although AGO2 levels are unchanged in *tsn1/tsn2* mutants, sRNA levels are higher in AGO2 complexes. U6 RNA was used to show equal loading. **c** TSN2 degrades AGO2-associated sRNAs. Phosphor images of 5′-[^32^P] miR393b* and 5′-[^32^P]-atTAS1b-siR374( + ) after incubation with TSN2 and GST–AGO2 result in degradation of 5′-[^32^P] miR393b* and 5′-[^32^P]-atTAS1b-siR374(+). Degradation of sRNAs is largely inhibited after GAR domain deletion, as shown in the incubation of TSN2 and sRNAs with GST:∆170 AGO2. **d** The *tsn1tsn2* double- mutant plants are more resistant to bacterial infection. Error bars represent standard deviation. Asterisks indicate mean values that differ significantly from wild-type (**P-valve < 0.01). Tubulin levels were used to show equal loading for all Western blots. In all panels, signal intensity was calculated using ImageJ

Given these results, it was of interest to determine how TSN proteins regulate AGO2 in RNAi. We first examined if TSN binding to methylated AGO2 promotes AGO2 degradation, but we found that neither the AGO2 protein levels nor the degradation rate of AGO2 were significantly altered in the *tsn1/tsn2* double mutant (Fig. 5a). We then examined whether TSN binding modulates AGO2 activity through sRNA loading and target silencing. We found that in the *tsn1/tsn2* mutant, AGO2 sRNA loading was increased (Fig. 5b), which silenced the target genes more efficiently than that in the wild-type control (Supplementary Fig. 12a and b).

Animal TSNs were recently shown to mediate the endonucleolytic decay of human miRNAs^47^. To determine how TSN binding reduces AGO2-associated sRNAs, and to test whether plant TSNs also degrade sRNAs, we incubated 5′-[^32^P]-labeled miR393b* or AtTAS1b-siR374(+) with recombinant HIS-tagged TSN proteins. This resulted in the elimination of each sRNA, indicating that plant TSNs also have sRNA degradation activity. When co-incubating labeled sRNAs with GST-tagged AGO2 and TSN proteins in the presence of PRMT5, AGO2-associated sRNAs were largely reduced (Fig. 5c). However, the TSN-mediated degradation of AGO2-associated sRNAs was largely reduced when the GAR domain was deleted (Fig. 5c and Supplementary Fig. 13). These results suggest that the methyl-arginine residues at the *N*-terminal region of AGO2 recruit TSN proteins to inhibit AGO2-mediated silencing through sRNA degradation. Moreover, the *tsn1/tsn2* mutant was more resistant to bacterial infection (Fig. 5d), which is consistent with increased level of AGO2-associated sRNAs. Thus, TSNs bind with arginine-methylated AGO2 and degrade AGO2-associated sRNAs.

## Discussion

Initiating immune responses only upon pathogen infection to prevent unnecessary fitness cost is critical for host growth and homeostasis. AGOs and the associated sRNAs are important regulatory components of host immunity, and these have evolved as part of sophisticated immune responses to defend against pathogen infection in plants^48,49^. While AGO2 positively regulates plant immunity^19,50–52^, it was unclear how the AGO2 protein is regulated when it is mostly needed during infection.

In this work, we identified PRMT5 as an arginine methylation writer and the Tudor-domain protein TSNs as an arginine methylation reader of AGO2. PRMT5 acts as a switch for arginine methylation of AGO2, which mediates dual regulation of the AGO2–RNAi pathway. We have shown that PRMT5 methylates arginine residues within the *N*-terminal GAR domain of AGO2 and promotes AGO2 degradation by the 26S proteasome. At the same time, methylated arginine residues of AGO2 allow for the binding of TSN proteins. Recruited TSNs degrade AGO2-bound sRNAs to prevent silencing of the corresponding target mRNAs.

Under normal growth conditions without bacterial infection, the AGO2-mediated RNAi pathway needs to be properly controlled to dampen plant immune responses. Arginine methylation by PRMT5 can contribute to inhibition of AGO2-mediated RNAi by suppressing the levels of both AGO2 protein and its bound sRNAs. However, upon bacterial infection, PRMT5 is downregulated. While further work would be needed to demonstrate the importance of this downregulation for regulation of AGO2-mediated immunity, it is plausible that reduced arginine methylation of AGO2 could promote immunity by increasing both accumulation of AGO2 and AGO2-associated sRNAs.

PRMT5 is likely regulated transcriptionally by defense-related transcription factors. Indeed, transcription factors WRKY75, MYB80, and heat stress transcription factor C-1 were found to be bound to the PRMT5 promoter in DNA-affinity purification sequencing datasets^53^. Future experimental validation and analysis will help elucidate the regulatory mechanism of PRMT5 upon pathogen infection.

Arginine methylation has been extensively studied on histones, transcription factors, and other regulatory proteins, which regulate genome organization and replication, cell cycle and cell differentiation, protein transcription, spliceosome assembly, and gametogenesis^28,37,54^. In animal systems, arginine methylation was observed only in the germline-specific PIWI proteins, a subfamily of AGOs that contain only 2–7 RG/GR repeats. Methylated arginine residues of PIWI proteins provide a binding dock for Tudor-domain proteins. Animal Tudor-domain proteins regulate RNA metabolism and function, including small RNA processing^47^, mRNA splicing and degradation, translational repression, and transcriptional silencing^43–46^. Arginine-methylated mouse MIWI protein binds to the Tudor-domain protein Tdrd6, and the methylated Drosophila PIWI protein Aubergine binds to Tudor protein, both in a PRMT5-dependent manner^55^. Although plants lack PIWI subfamily proteins, plants have evolved several AGO proteins that contain a large number of RG/GR repeats, such as AGO2, AGO3, and AGO1 in Arabidopsis, allowing PRMT5-mediated arginine methylation and subsequent Tudor protein binding. These results suggest that arginine methylation-mediated AGO association with Tudor proteins is an evolutionary conserved regulatory mechanism of RNAi, although the output of the AGO–Tudor binding may vary in different tissues and different organisms. Unlike the situation in plants, the interaction of arginine-methylated MIWI/PIWI with Tudor proteins in animal germline cells often do not alter the levels of MIWI/PIWI proteins and the associated sRNAs, but rather change the subcellular localization of the MIWI/PIWI proteins or the abundance of certain classes of sRNAs, which is important for gametogenesis^43–46^. This study has demonstrated that arginine methylation can regulate Arabidopsis AGO2. We believe that in-depth functional analysis of dynamic PTMs of key regulatory proteins of RNAi pathways will provide substantial understanding of sophisticated and fine-tuned regulation of RNA-silencing pathways and host immunity.

## Methods

### Plant growth conditions

*Arabidopsis thaliana* accession Col-0 was the background for all mutants except *tsn1tsn2* (*tsn1* is Wassilewskija (WS) background and *tsn2* is Col-0 background). The *prmt5-1* mutant is a T-DNA insertion knockout line and was characterized and reported previously^56,57^. For pathogen assays and biochemical experiments, candidate seeds were grown in soil side-by-side at 23 °C for 4 weeks under short-day periods (12 h of light followed by 12 h of darkness).

### Constructs and transgenic plants

The *PRMT5* coding sequence was fused with a FLAG-tag at the *N*-terminal region and cloned into the pZP vector to produce Flag:PRMT5 driven by two cauliflower mosaic virus (CaMV) 35S promoters. The HSP90 coding sequence was amplified and cloned into pENTR using pENTR^™^/SD/D-TOPO^™^ Cloning Kit (Invitrogen). The pEarly-202 destination construct was used to form Flag:HSP90 by Gateway LR Clonase II Enzyme Mix (Invitrogen). Flag:Memb12 was constructed as previously described^19^. In addition, the *AGO2* coding sequence was fused with the three HA tags at the 5′ region in pMDC32 vector to yield HA:AGO2 driven by the CaMV35S promoter. The *Δ*59 (deletion of the first 59 aa residues of AGO2 as indicated in Supplementary Fig. 3a), *Δ*62 (deletion of the middle 62 aa residues of AGO2 as indicated in Supplementary Fig. 3a), and *Δ170* (deletion of the total GAR domain of AGO2 as indicated in Supplementary Fig. 3a) were also fused to HA tags and cloned into the pMDC32 vector under the control of 35S promoter or native promoter to yield *HA:Δ59AGO2*, *HA:Δ62AGO2, HA:Δ170AGO2*, and *pAGO2*:*3HA:Δ59 AGO2*, respectively. *PRMT5*, *TSN1,* and *TSN2* coding sequences were cloned into the pEarly-Gate 203 binary vector with a c-Myc tag fusion using Gateway Cloning strategy. The *pAGO2*:*3HA:Δ59 AGO2* transformants were made by transforming the *pAGO2*:*3HA*:*Δ59 AGO2* into *ago2-1* mutants and selected on MS medium supplemented with hygromycin B (30 mg L^−1^).

### Protein pull-down and mass spectrometry analysis

The transgenic plants p*AGO2*:*3HA*:*AGO2* and p*AGO7*:*gus* treated with *Pst* (*avrRpt2*) were used for co-IP experiments to identify AGO2-associated proteins. Anti-HA antibody was used for immunoprecipitation in both lines (Exp1: treated with p*AGO2*:*3HA*:*AGO2*; Exp2: treated with p*AGO7*:*gus*). The pull-down samples were denatured by heating to 95 °C in Laemmli loading buffer and then separated on a 12% SDS-PAGE gel with a 4% stacking gel. The resulting gel was equally cut into five slices. The proteins in each individual gel slice were reduced in-gel with dithiothreitol and alkylated with iodoacetamide. The processed proteins were subsequently digested with trypsin (Promega, Madison, WI) at 37 °C overnight. Subsequently, peptides were extracted from gels with a solution containing 5% acetic acid in H_2_O and then CH_3_CN/H_2_O (1:1, v/v).

Online LC–MS/MS analyses were conducted on an LTQ-Orbitrap Velos mass spectrometer equipped with a nanoelectrospray ionization source and coupled with an EASY n-LCII HPLC system (Thermo, San Jose, CA). HPLC separation was carried out automatically using a homemade trapping column (150 μm × 50 mm) and a separation column (75 μm × 200 mm, packed with ReproSil-Pur C18-AQ resin, 3 μm in particle size and 100 Å in pore size). The peptides were separated using a 120-min linear gradient of 2–40% CH_3_CN in 0.1% formic acid at a flow rate of 230 nL min^−1^. All MS/MS spectra were acquired in a data-dependent scan mode, where one full-scan MS (from *m/z* 350 to 2000) was followed with 20 MS/MS scans at a normalized collision energy of 35%. The identification and quantification of AGO2-associated proteins were achieved by searching the LC–MS/MS data using Maxquant Version 1.2.2.5^58^. The search was performed with the tolerances in mass accuracy of 10 ppm and 0.6 Da for MS and MS/MS, respectively. Candidates from Exp1 not in Exp2 were selected using an LFQ intensity value. Low PEP value (less than 0.1) candidate peptide sequences were considered as the most likely candidates to bind AGO2.

### Transient expression in *N. benthamiana*

*N. benthamiana* plants were grown under the same conditions as Arabidopsis plants before *Agrobacterium*-mediated transient expression experiments. *Agrobacterium GV3101*, which was used for transformations of each construct, was grown in Luria–Bertani broth with the corresponding antibiotic selections at 28 °C overnight. Cell cultures were resuspended in infiltration buffer (10 mM 2-(*N*-morpholino) ethanesulfonic acid (MES) and 10 mM MgCl_2_, 200 μM acetosyringone) at OD600 nm = 0.8 and incubated at room temperature for at least 4 h before infiltration. After 2 days, the infiltrated leaves were collected for BiFC and co-IP experiments.

### BiFC

The *AtAGO2* and *AtAGO10* ORF fragments were PCR-amplified and inserted into the n-Venus vector pSAT1-nVenus-N to generate n-Venus-AGO2 using SacI/SalI sites and n-Venus-AGO10 using Xho1/BamH1, respectively. Simultaneously, the total PRMT5 and HSP90 fragments were PCR-amplified and inserted into the c-CFP vector pSAT4-cCFP-N to generate cCFP-PRMT5 and cCFP-HSP90 using SacI/SalI or PstI/KpnI sites, respectively. All constructs were transformed into *Agrobacterium* and co-infiltrated into *Nicotiana benthamiana* leaves. After culturing for 60–70 h, the fluorescent signals from leaf epidermal cells were detected using a Zeiss SP5 confocal microscope. The channel specifications used were as follows: Argon laser, excitation is active at 488 nm; transmission 8%; main beam splitter 1: 488/543/633; beam splitter 2: 545; BP 500–530IR; detector gain: 620; amplifier offset: −0.1.

### Bacterial infection

Bacterial growth assays were performed as described^19^. For infection, bacteria were first grown on a PAF medium plate at 28 °C for 2 days before being resuspended in 10 mM MgCl_2_ solution for infiltration. The antibiotic-selection concentrations used for *Pst (EV)* and *Pst* (*avrRpt2*) were 25 μg ml^−1^ rifampicin and 50 μg ml^−1^ kanamycin. For protein extraction, plants were inoculated with *Pst* strains at a concentration of 1 × 10^7^ cfu ml^−1^. For bacterial growth assays, 5 × 10^5^ cfu ml^−1^ was used. Infected leaf samples were collected on day 0 and day 3. At least 10 leaf disks were collected for each experimental sample. Student’s *t* test was used to determine significant differences between mutants and control plants.

### Protein extraction and analysis

Leaves were grounded in liquid N_2_ and collected by protein extraction buffer, which contained 20 mM Tris-HCl, pH 7.5, 300 mM NaCl, 5 mM MgCl_2_, 0.5% (v/v) Nonidet P-40, 5 mM DTT, and protease inhibitor cocktail (Roche, one tablet for 50 ml). The intercellular fluid was collected from the same amount of tissue by centrifuging leaves at 1500 *g* for 5 min as described previously^19^. For input protein, the supernatant was added to loading buffer (125 mM Tris-HCl, 4% (w/v) SDS, 20% (v/v) glycerol, 2% (v/v) 2-mercaptoethanol, and 0.05% (w/v) bromophenol blue), and boiled at 110 °C for 5 min, followed by cooling on ice and loading on an SDS-PAGE gel for western-blot analysis. For co-IP experiments, the supernatant was added to 25 μL (per gram tissue) of Protein A-Agarose beads to pre-equilibrate the protein extract. After 1 h of rotation at 4 °C, the supernatant was collected by centrifuging at 200 *g* for 1 min. In total, 25 μL of the corresponding anti-tag Affinity Matrix or antibody with Protein A-Agarose beads was then added. After shaking the samples for 2 h at 4 °C, the beads were collected by 200 *g* centrifugation for 1 min and washed three times with washing buffer (20 mM Tris-HCl, pH 7.5, 300 mM NaCl, 5 mM MgCl_2_, 0.5% (v/v) Triton X-100, 5 mM DTT, and protease inhibitor cocktail). The beads were then used for further western-blot analysis or RNA extraction. The proteins from the same volume of each sample were analyzed by western-blot analysis using α-tubulin as a loading control and the relative amount (RA) value measurements.

In all the western blotting analysis, the anti-HA horseradish peroxidase (HRP)-conjugated antibody (Santa Cruz Biotechnology, Cat#: sc-7392, RRID: AB_627809, 1:1000 dilution) was used to detect the HA-tagged proteins; the anti-Myc HRP (Thermo Fisher Scientific, Cat #: AHO0062, RRID: AB_2536303, 1:2000 dilution) was used to detect the c-Myc-tagged proteins; the monoclonal ANTI-FLAG(R) M2-Peroxidase (HRP) antibody produced in mouse (Sigma-Aldrich, Cat#: A8592, RRID: AB_439702, 1:2000 dilution) was used to detect the Flag-tagged proteins. The anti-green fluorescent protein antibody (Roche, Cat#:11814460001, RRID: AB_390913, 1:2000 dilution) was used to detect the YFP-fused proteins; the monoclonal anti-α-tubulin antibody produced in mouse (Sigma-Aldrich, Cat#: T6074, RRID: AB_477582, 1:5000 dilution) was used to detect the α-tubulin level as a loading control. The goat anti-Ig polyclonal antibody HRP-conjugated (BD Biosciences, Cat#: 554002, RRID: AB_395198, 1:5000 dilution) was used as a secondary antibody when detecting tubulin and the YFP-fused proteins. The rabbit polyclonal anti-AtAGO2 (1:1000 dilution) and the rabbit polyclonal anti-MEMBER12 (1:1000 dilution) were made and published in molecular cell (2011); the rabbit polyclonal anti-TSN (1:1000 dilution) and rabbit polyclonal anti-PRMT5 (1:1000 dilution) were provided by other labs that were mentioned in the section Acknowledgements. The goat anti-rabbit IgG—H&L Polyclonal antibody, HRP-conjugated, (Abcam, Cat#: ab6721, RRID: AB_955447, 1:5000 dilution) was used as the secondary antibody to detect these native antibodies.

### RNA immunoprecipitation and analysis

RNA extraction and immunoprecipitation were performed as described previously^19^. In total, 20 g of leaf tissue from 4-week-old plants were collected and grounded with liquid N_2_. The intercellular fluid was used for examining RNAs co-immunoprecipitated with AGO2 using an AGO2 antibody. The RNA from AGO2-IP and from total extracts was isolated by TRIzol and used in northern and real-time PCR assays. The sequences of probes and primer pairs are listed in Supplementary Table 1.

### GST pull-down assays

Matrix-bound GST-fusion proteins (GST:AGO2, GST:N170 AGO2, and GST:Δ170 AGO2) were incubated with *E. coli* extracts containing HIS:PRMT5 at 4 °C for 2 h with constant gentle mixing. The mixtures were centrifuged at 5000 *g* for 5 min, and the pellet was washed extensively with buffer (20 mM Tris/HCl, pH 7.5, 150 mM NaCl, 5 mM EDTA, 1% Nonidet P-40, and 0.5% sodium deoxycholate, with 1 mM phenylmethane sulfonyl fluoride (PMSF) and 0.5 mM dithiothreitol added fresh before use). After the final wash, the pellet was resuspended in an equal volume of 2x SDS loading buffer and separated by 12% SDS-PAGE gel. The anti-GST HRP (Santa Cruz Biotechnology, Cat#: sc-138, RRID: AB_627677, 1:1000 dilution) was used to detect the GST-tagged recombinant proteins; the mouse anti-histidine monoclonal antibody (R&D Systems, Cat #: MAB050H; RRID: AB_357354, 1:2000 dilution) was used to detect the HIS-tagged recombinant proteins.

### Methylation assays in vivo and in vitro

The fusion proteins GST:PRMT5, GST:AGO2, GST; N170 AGO2, and GST:Δ170 AGO2 were expressed in *Escherichia coli* strain BL21 cells and affinity-purified with glutathione–sepharose beads (Amersham Biosciences). The candidate genes were cloned into the pDest 15 vector and transformed into BL21 strains. The *E. coli* strain was cultured in liquid LB medium at 37 °C until the O.D._600_ value = 0.6. 0.1 mM isopropyl β-D-1-thiogalactopyranoside (IPTG) was added for inducing expression for all recombinant proteins at 16 °C for 16 h. The cell cultures were collected by centrifugation at 4000 *g* for 10 min and resuspended in PBS buffer (pH 7.4) with 0.1% Triton X-100 and 0.1 mM PMSF. Soluble proteins were cleared by centrifugation, purified with 100 µl of glutathione–sepharose beads, and washed twice in PBS buffer. For in vitro histone methyltransferase assays, purified proteins were incubated with GST–PRMT5 at 30 °C for 1 h in the presence of [^3^H] *S*-adenosyl-L-methionine (SAM). Labeled proteins were separated by SDS/15% PAGE and visualized by autofluorography for 48 h. The anti-sDMA antibody*, SYM10*, and anti-aDMA antibody, anti-ASYM24 were used to detect the in vivo amount of methylated AGO2 proteins by western-blot analysis. The anti-dimethyl-arginine, symmetric (SYM10) antibody (Millipore, Cat#: 07–412; RRID: AB_310594, 1:1000 dilution) and anti-dimethyl-arginine, asymmetric (ASYM24) antibody (Millipore, Cat#: 07–414, RRID: AB_310596, 1:1000 dilution) were used to detect the in vivo amount of methylated AGO2 proteins by western-blot analysis.

### In vitro nuclease assays

Synthetic single-stranded sRNAs (Integrated DNA Technology) were 5´-end labeled using γ-[^32^P] ATP (Perkin-Elmer) and T4 polynucleotide kinase (New England Biolabs). For nuclease assays, labeled miRNAs (100–200 fmol) were incubated for 2 h at 37 °C with *E. coli*-produced GST–TSN (1–2 pmol) in 15 µl of nuclease reaction buffer (50 mM HEPES–KOH, pH 7.5, 5 mM CaCl_2_, 100 mM NaCl, and 1 mM ATP). Reactions were terminated by adding 1 µl of proteinase K (Thermo Fisher Scientific) for 5 min at 37 °C, and electrophoresed in 6 M urea, 15% polyacrylamide gels, and visualized using a Typhoon Phosphorimager (GE Biosciences).

### Assembly of AGO2-loading [^32^P] sRNA nuclease assays

Recombinant GST:AGO2 and GST:Δ170 AGO2 proteins were incubated for 1 h at 37 °C with a 5´-[^32^P]-miR393b*:5´-[P]-miR393 duplex in loading buffer [15 mM HEPES–KOH, pH 7.4, 54 mM potassium acetate, 1 mM magnesium acetate, 8.5 mM DTT, 30 mM creatine phosphate, 0.4 U µl^−1^ RNaseOUT (Thermo Fisher Scientific), 1.2 mM ATP, 0.3 mM GTP, and 90 µg ml^−1^ creatine kinase]. GST:AGO2/GST:Δ170 AGO2 was immunoprecipitated by GST beads and washed using washing buffer [50 mM Tris-HCl, pH 7.4, 150 mM NaCl, and 0.05% NP40]. The AGO2-loaded [^32^P]-labeled RNA was incubated for 2 h with 50 µl of nuclease reaction buffer with or without 5 pmol of HIS–TSN at 37 °C.

### Reporting summary

Further information on experimental design is available in the Nature Research Reporting Summary linked to this article.

## Supplementary information

Supplementary Information

Description of Additional Supplementary Files

Supplementary Data 1

Reporting Summary

Source Data

## Data Availability

The mass spectrometry dataset was uploaded in PeptideAtlas. The Accession code is PASS01211. The authors declare that all the data supporting the findings of this study are available within the article. All lines and material generated in this study are available from the corresponding author upon request. The source data underlying Figs. 1b, 1c, 1e, 2a, 2c-e, 3b-g, 4a-d and 5a-d and Supplementary Figures 1b, 3b, 5, 6a, 7, 8a, 9a, 11a-c, 12a and 13 are provided as a Source Data PDF file.
