# Supplementary material for: Dual regulation of Arabidopsis AGO2 by arginine methylation
Source: Nat Commun. 2019 Feb 19;10:844. doi: 10.1038/s41467-019-08787-w (PMC6381116; doi:10.1038/s41467-019-08787-w)
Supplement: Supplementary file 1 — Supplementary Information [file 41467_2019_8787_MOESM1_ESM.pdf]

Dual regulation of Arabidopsis AGO2 by arginine methylation

Po Hu et al.

Supplementary Information

This file contains Supplementary Figure 1 -13 with figure legends and Supplementary Table 1 (The primers list).

Supplementary figure 1

a

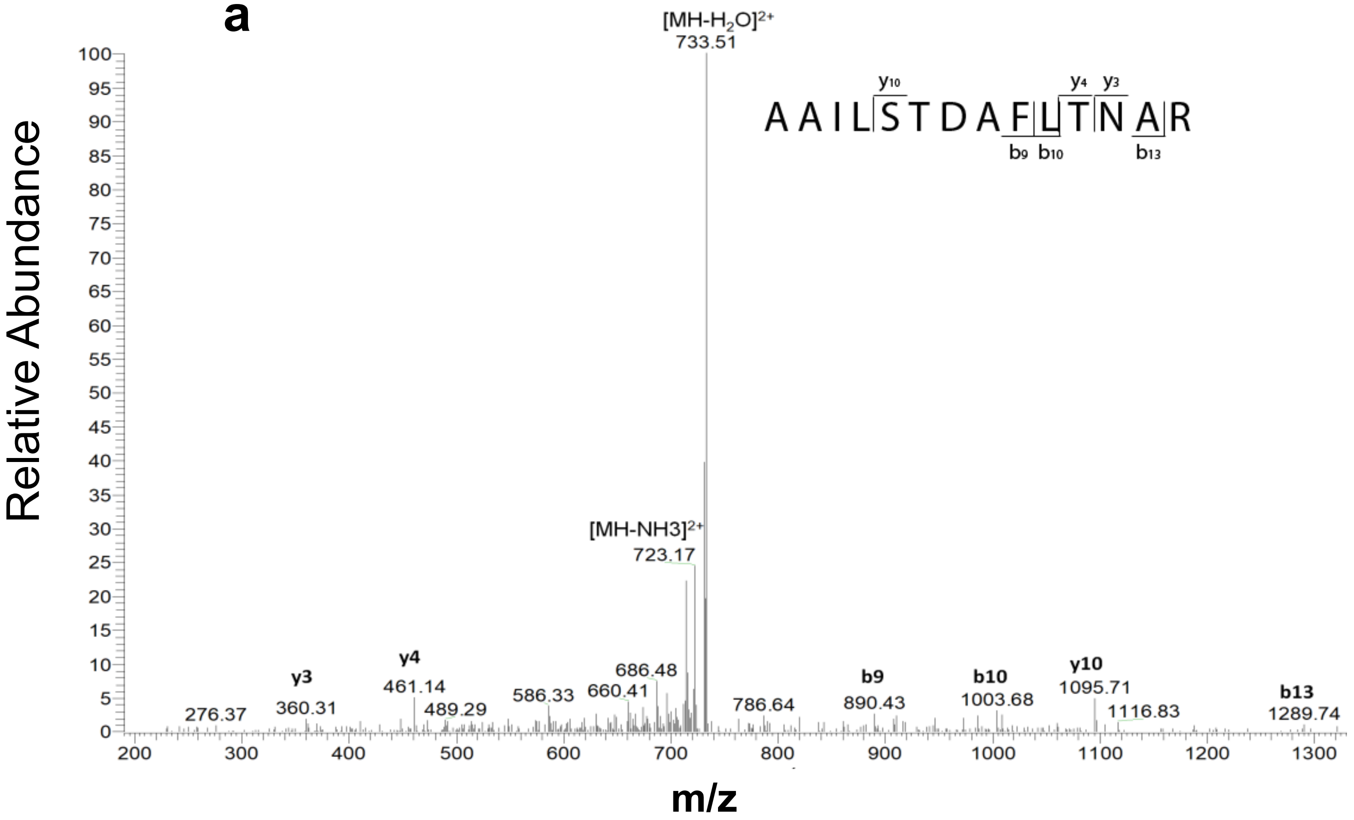

b

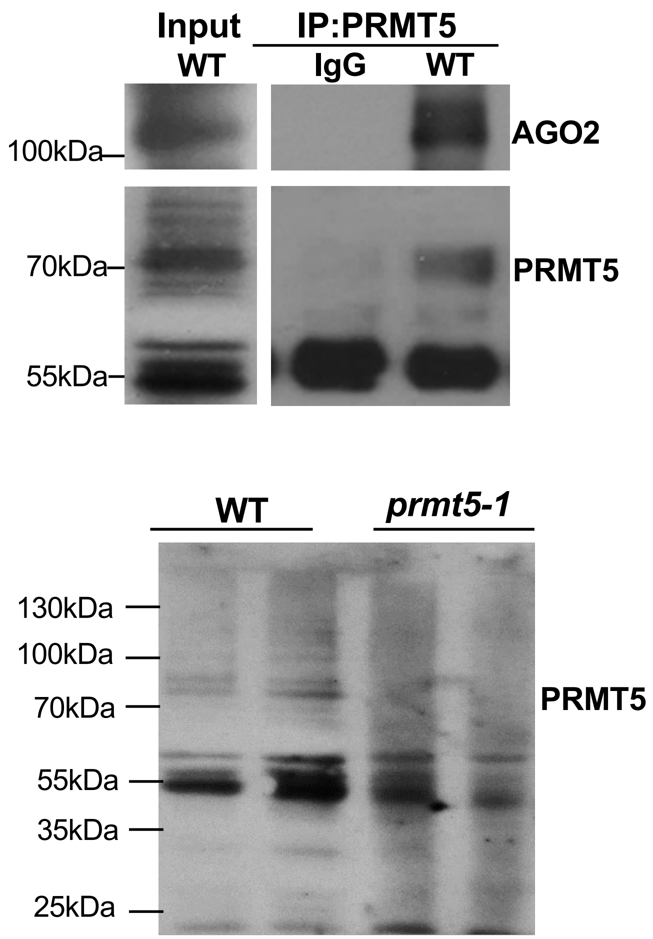

Supplementary figure 1. PRMT5 interacts with AGO2

(a) Representative MS/MS spectra of other PRMT5 peptides in addition to the one presented in Figure 1a obtained from the HA:AGO2 pull down experiment. The peptide sequence was assigned with a single letter abbreviation based on the fragmented ions observed for the peptide segments. (b) The interaction between PRMT5 and AGO2 were detected in *Arabidopsis* wild type plants. Protein complexes were immunoprecipitated using anti-PRMT5 antibody, and the immunoblots were probed with native antibodies against AGO2 or PRMT5. The PRMT5 antibody was validated using the *prmt5-1* mutant shown in the bottom panel. The PRMT5 protein is about 72kDa. The AGO2 protein is about 113kDa.

Supplementary figure 2

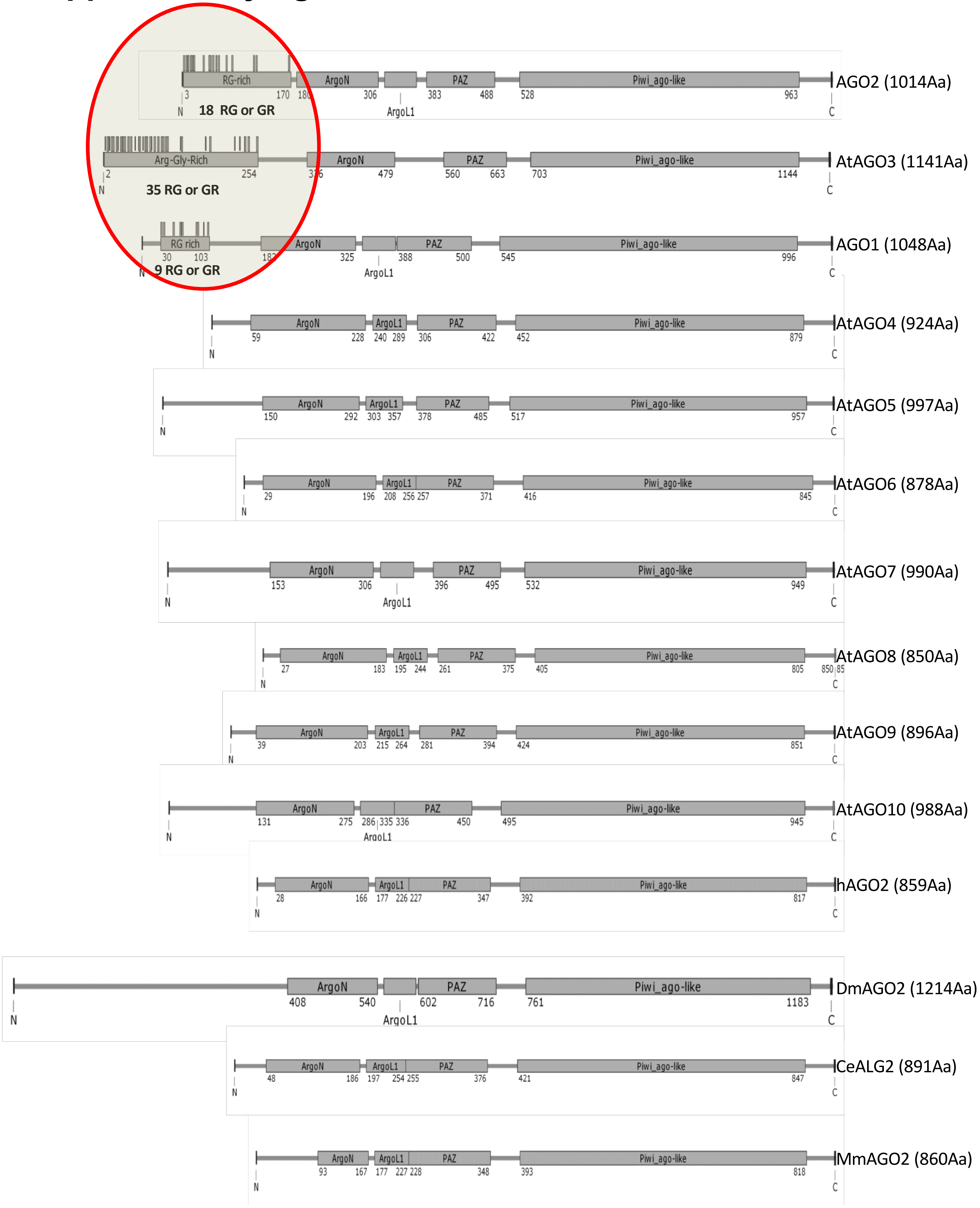

Supplementary figure 2. Glycine-arginine-rich (GAR) regions were found in only three Arabidopsis AGO proteins (in the red oval).

Supplementary figure 3

a

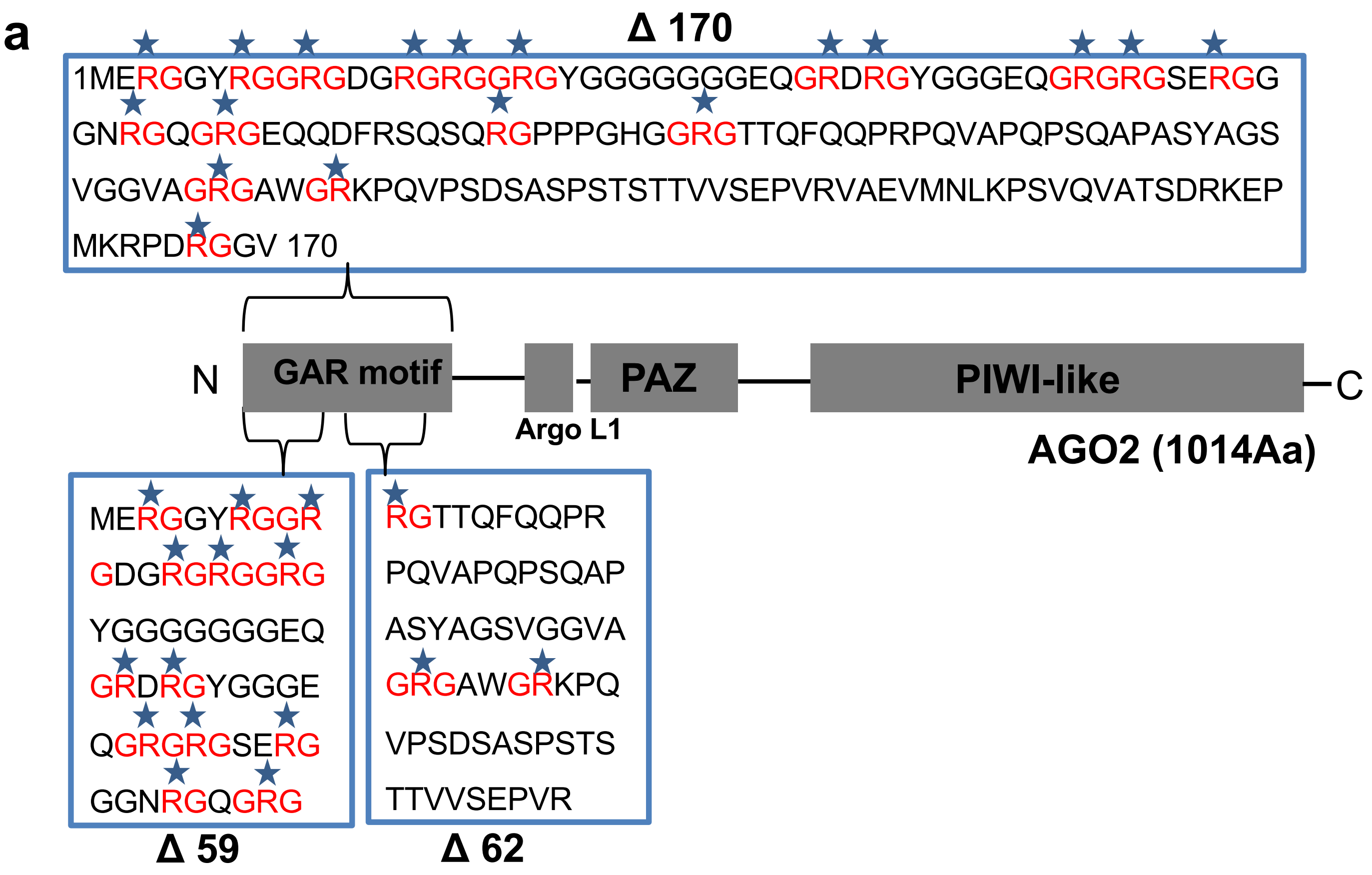

b

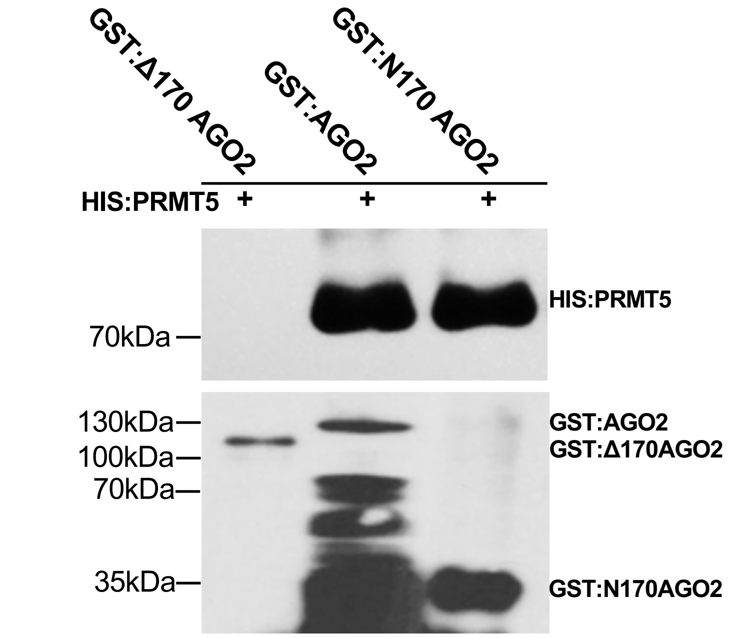

Supplementary figure 3. AGO2 N-terminal GAR region is essential for arginine methylation by PRMT5. (a) The amino acid sequence of Δ 59, Δ62 and Δ170 peptides from the N-terminal region of AGO2. The RG/GR repeats were marked in red and by stars. (b) The bait–prey protein pairs were detected in the GST pull-down assay, which indicated that HIS:PRMT5 bind to AGO2 at its N-terminal region.

Supplementary figure 4

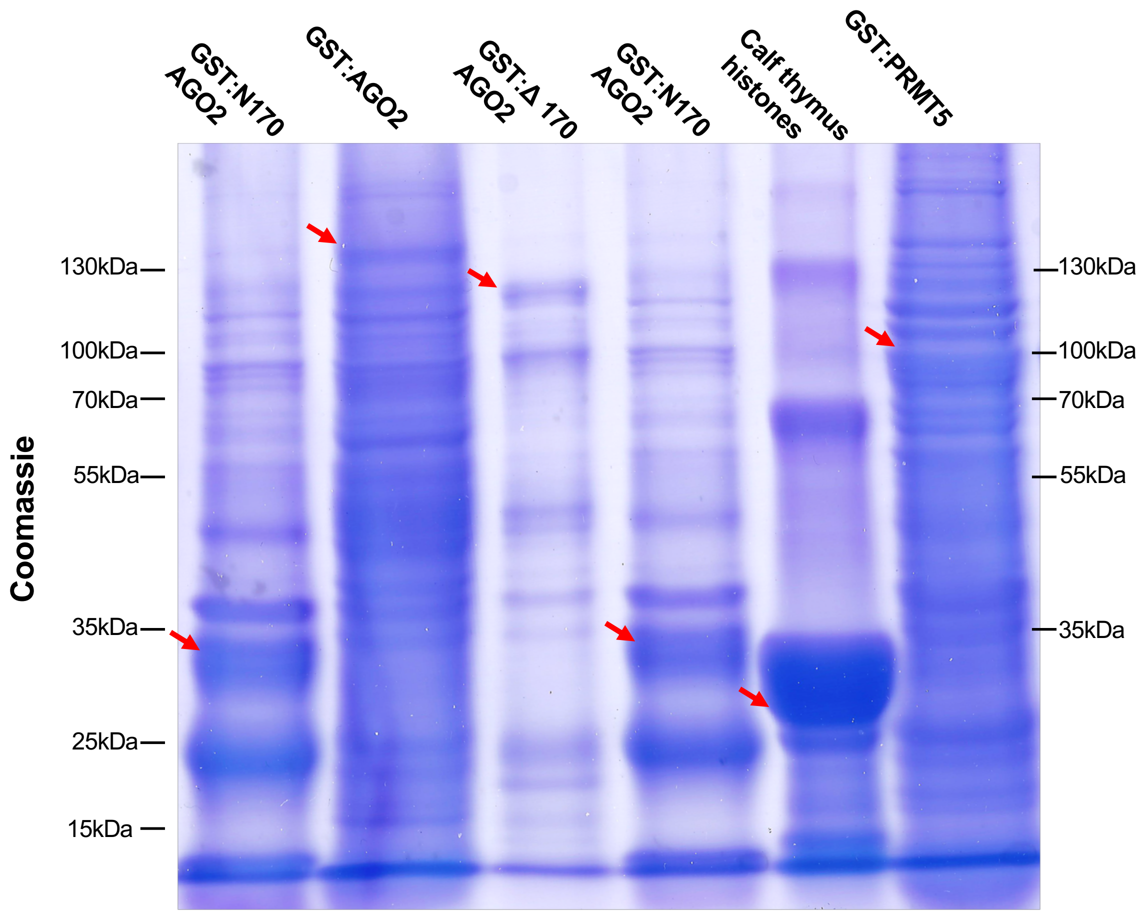

Supplementary figure 4. Recombinant GST:AGO2, GST:N170 AGO2, GST: Δ170 AGO2, GST:PRMT5 were expressed in *E.coli* (BL21). Coomassie blue staining showed the corresponding proteins of each panel related to Figure 2b. The GST:PRMT5 was loaded at the right. The red arrows indicated the corresponding bands. The molecular weight of these proteins are: GST:N170 AGO2-33 kDa; GST:AGO2-132 kDa; GST:Δ170 AGO2-115 kDa; GST:PRMT5-100kDa.

Supplementary figure 5

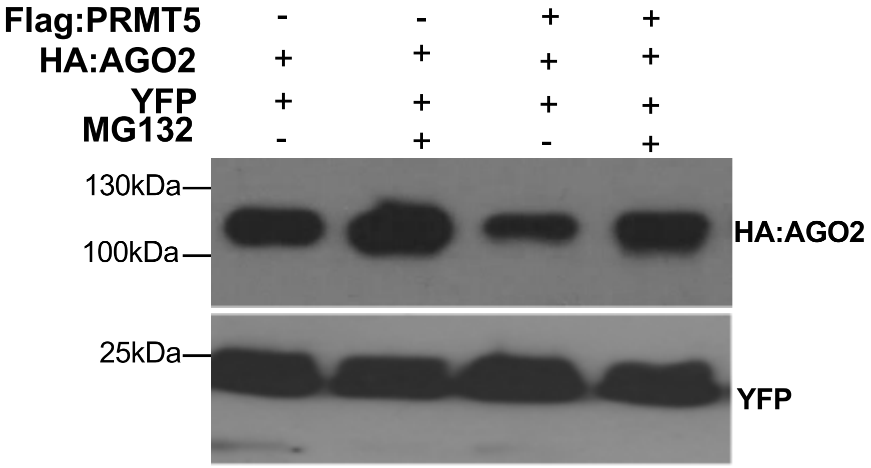

Supplementary figure 5. Degradation of AGO2 is dependent on PRMT5 and 26S proteasome in N. *benthamiana* transient expression assay. MG132 inhibits 26S proteasome-mediated degradation. N. *benthamiana* leaves were infiltrated with the same amount of bacteria carrying YFP construct for transient expression as a control. The MG132 was infiltrated at the same time with the Agrobacteria.

Supplementary figure 6

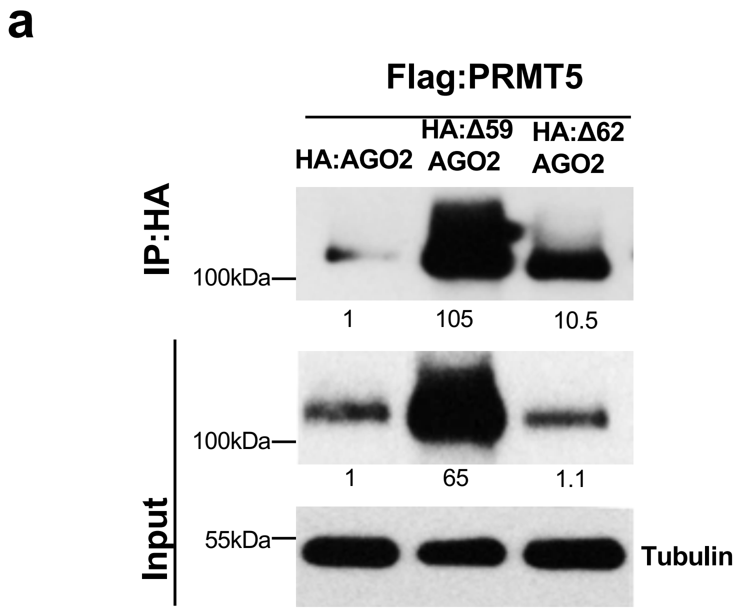

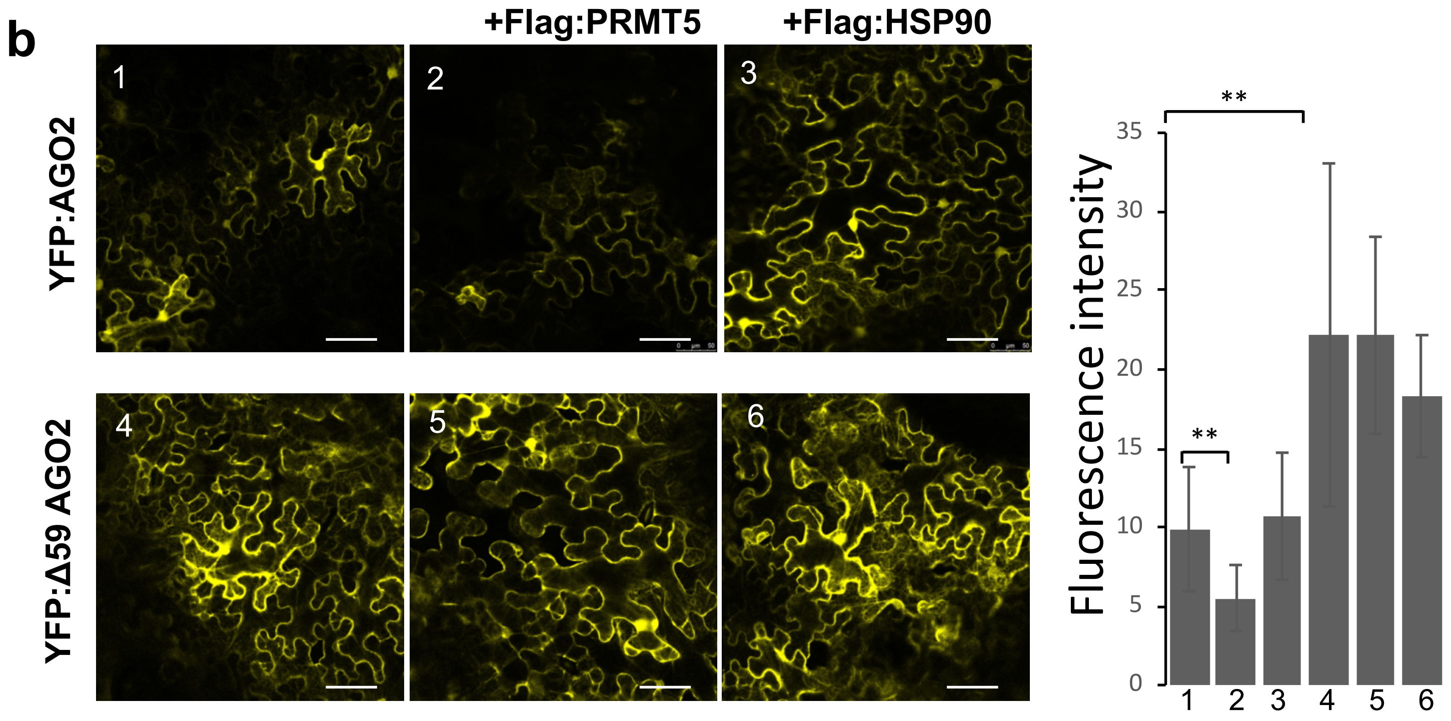

Supplementary figure 6. The degradation of AGO2 is dependent on the N-terminal GAR region.

(a) Deletion of the GAR motif enhances AGO2 protein stability. The protein level of full-length AGO2 (HA:AGO2) and truncated AGO2 (HA:Δ*59 AGO2 and HA:*Δ*62 AGO2*) were tested using anti-HA antibodies after N. *benthamiana* transient expression. Tubulin was used as an internal control for equal loading. (b) The fluorescence signal of YFP:AGO2 is weakened after being co-expressed with PRMT5, but the level of YFP:Δ59 AGO2 remains high when being co-expressed with PRMT5. Scale bar, 150μm. 1. YFP:AGO2; 2. YFP:AGO2+Flag:PRMT5; 3. YFP:AGO2 +Flag:HSP90; 4. YFP:Δ59 AGO2; 5.YFP:Δ59 AGO2 +Flag:PRMT5; 6. YFP:Δ59 AGO2 +Flag:HSP90. The fluorescence intensity was measured in the right chart. Error bars represent standard deviation from 10-13 microscope pictures. Asterisks indicate mean values that differ significantly from YFP:AGO2 (**P-valve<0.01).

Supplementary figure 7

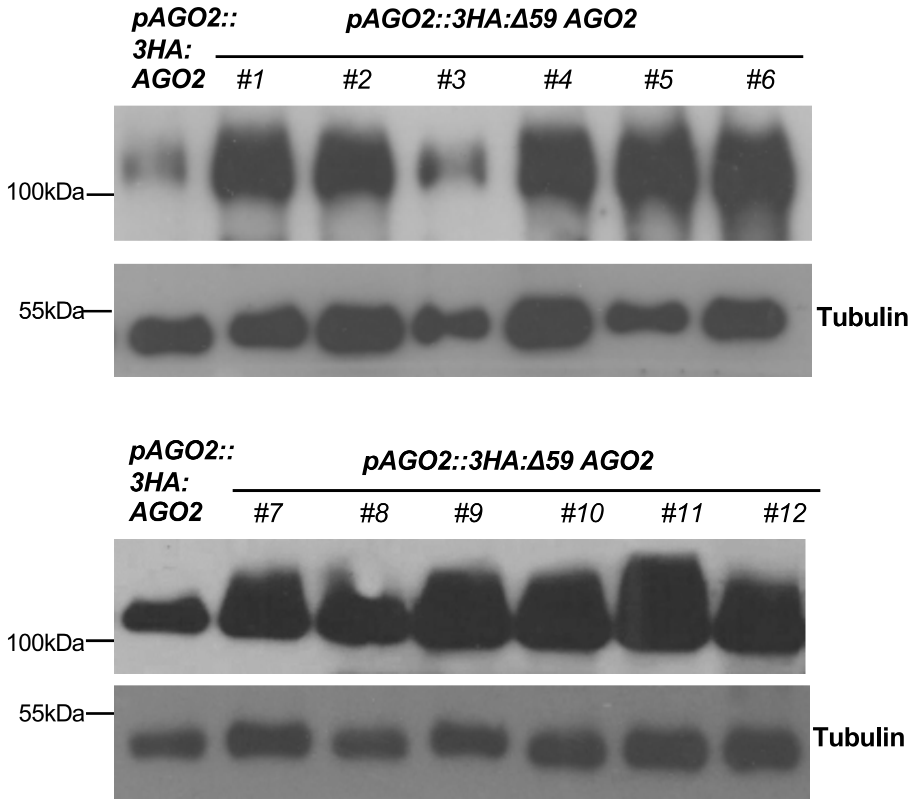

Supplementary figure 7. The AGO2 protein levels were examined in the *pAGO2::3HA:Δ59 AGO2* transgenic lines using anti-HA antibodies. *pAGO2::3HA:AGO2* served as a control for 3HA:AGO2 expression. The *pAGO2::3HA:Δ59 AGO2-1* and *pAGO2::3HA:Δ59 AGO2-2* lines were selected for the pathogen assay. The *pAGO2::3HA:Δ59 AGO2-1* was selected for subsequent experiments and is identified as *pAGO2::3HA:Δ59 AGO2*.

Supplementary figure 8

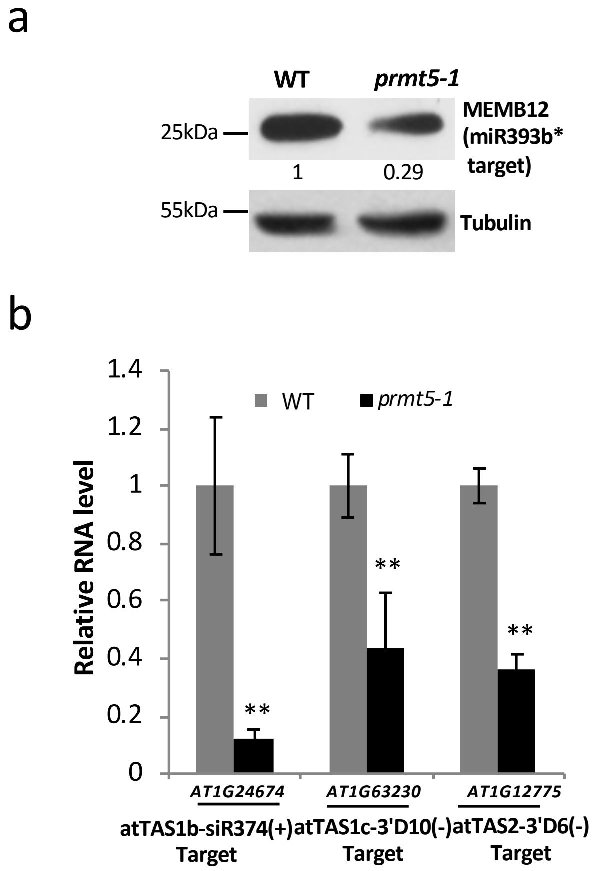

Supplementary figure 8. Expression of the AGO2-associated sRNA targets was measured by Western blot (a) or quantitative RT-PCR (b) in the wild type (WT) and *prmt5-1*. The associated protein analyzed was the miR393b* target MEMB12; and the associated mRNA targets include the atTAS1b-siR374(+) target *AT1G24674*, the atTAS1c-3'D10(-) target *AT1G63230,* and the atTAS2-3'D6(-) target *AT1G12775*. Error bars represent standard deviation. Asterisks indicate mean values that differ significantly from wild type (*P-value<0.05, **P-valve<0.01).

Supplementary figure 9

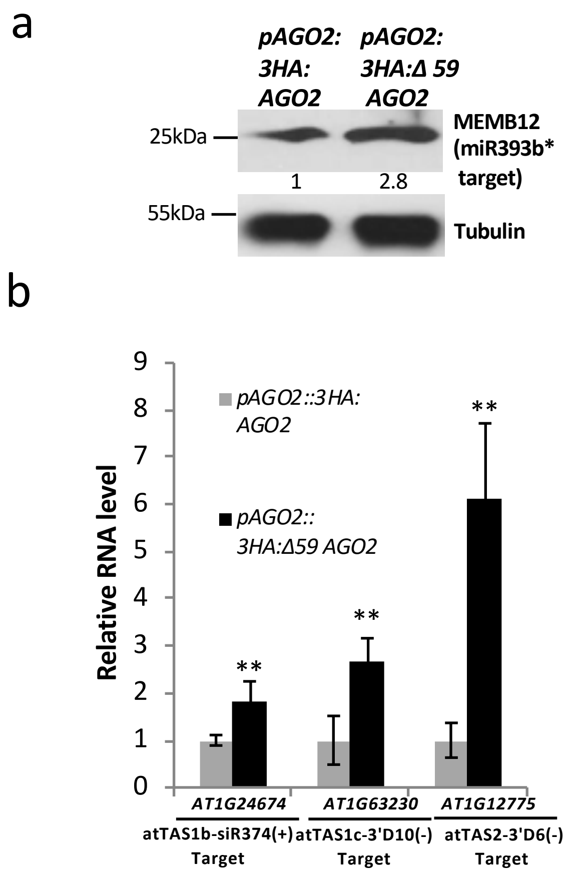

Supplementary figure 9. Expression of the AGO2-associated sRNA targets was measured by Western blot (a) or quantitative RT-PCR (b) in *pAGO2::3HA:AGO2* and *pAGO2::3HA:Δ59 AGO2* lines. The miR393b* target MEMB12, the atTAS1b-siR374(+) target *AT1G24674,* the atTAS1c-3'D10(-) target *AT1G63230,* and the atTAS2-3'D6(-) target *AT1G12775* were analyzed. Error bars represent standard deviation. Asterisks indicate mean values that differ significantly from wild type (**P-valve<0.01).

Supplementary figure 10

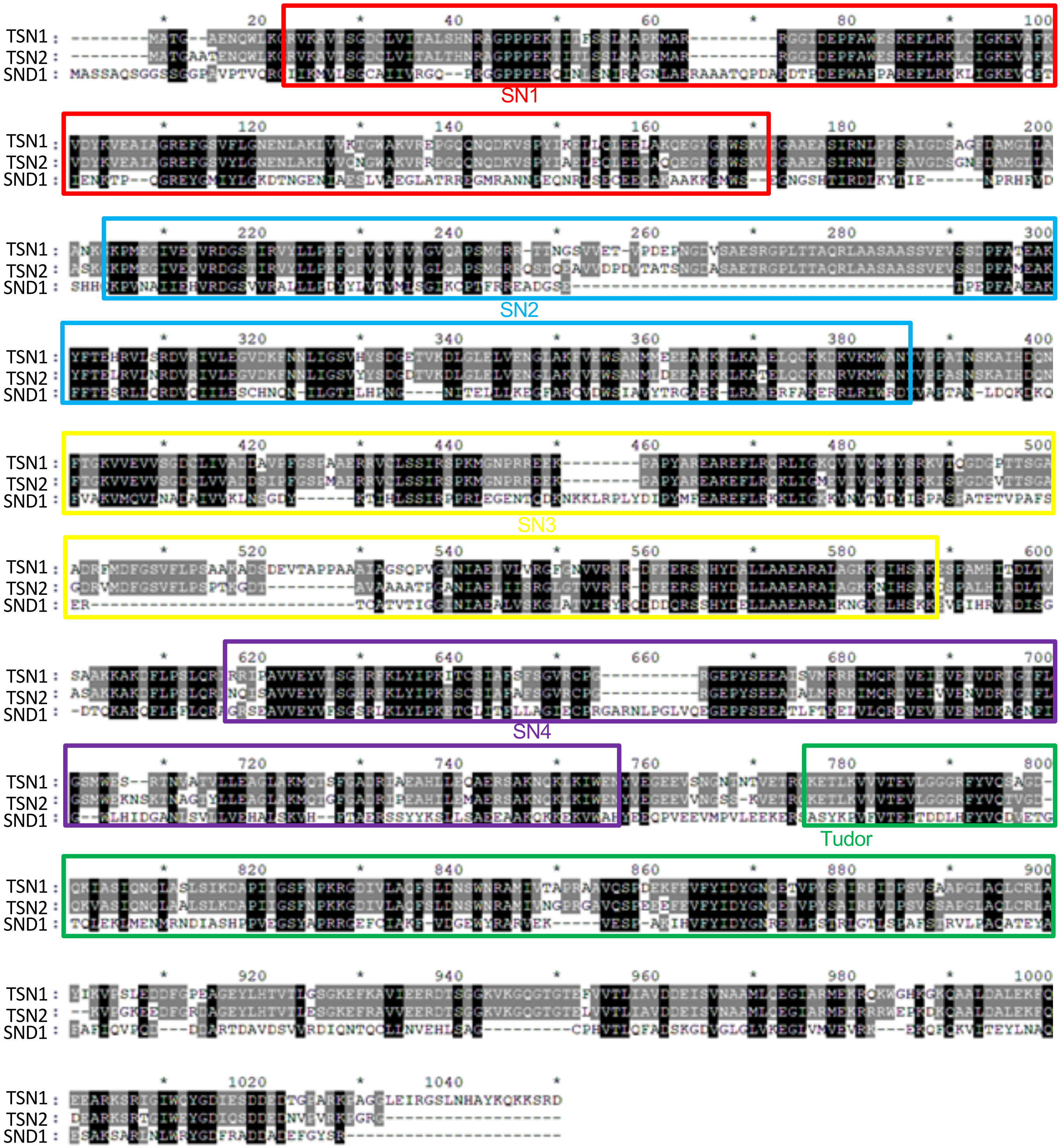

Supplementary figure 10. Amino acid sequence alignment of *Arabidopsis* TSN1,TSN2 and Human SND1. Identical amino acids are marked in black and similar amino acids are marked in grey. The four SN domains were boxed by colored rectangles: SN1-red; SN2-blue;SN3-yellow; SN4-purple. The Tudor domain was boxed in green.

Supplementary figure 11

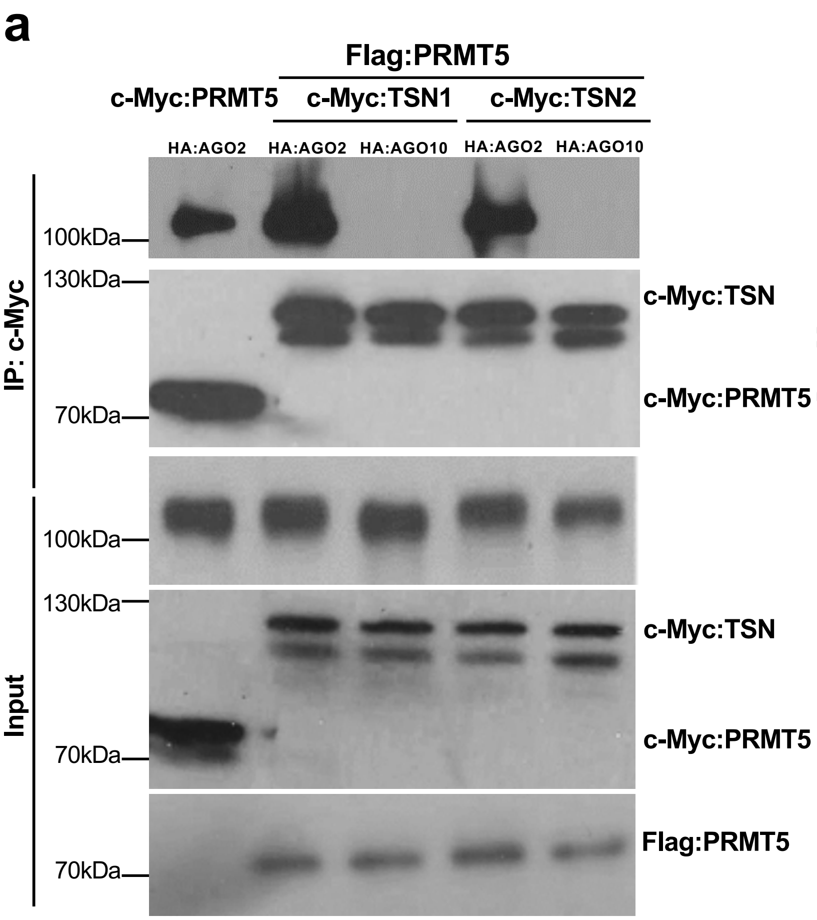

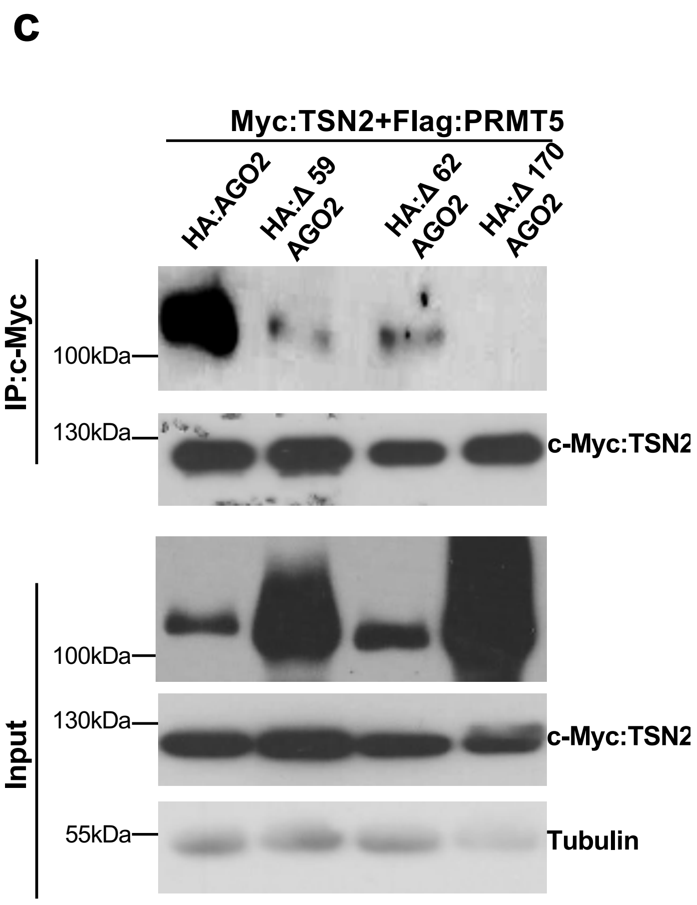

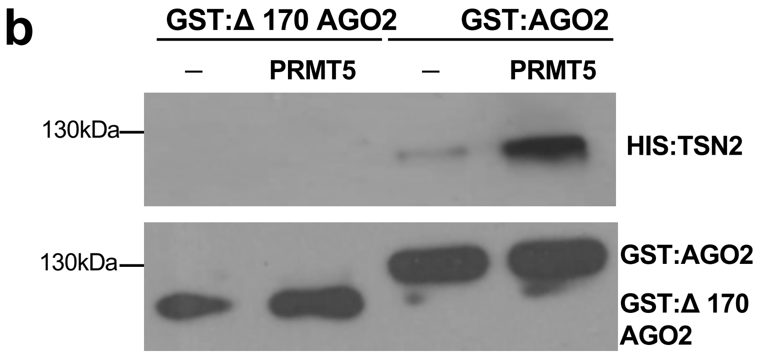

Supplementary figure 11. *Arabidopsis* TSN proteins interact with AGO2

(a) TSN1 and TSN2 bind to the AGO2 protein but not AGO10 in *N. benthamiana* transient expression assay*.* Complexes were immunoprecipitated using anti-Myc antibodies and immunoblotted with anti-HA antibodies. Myc:PRMT5 serves as positive control and HA:AGO10 as negative control. (b) TSN2 preferentially binds to methylated AGO2 *in vitro*. (c) Deletion of the N-terminal region (Δ 59, Δ 62 and Δ 170) largely reduced the binding between AGO2 and TSN2.

Supplementary figure 12

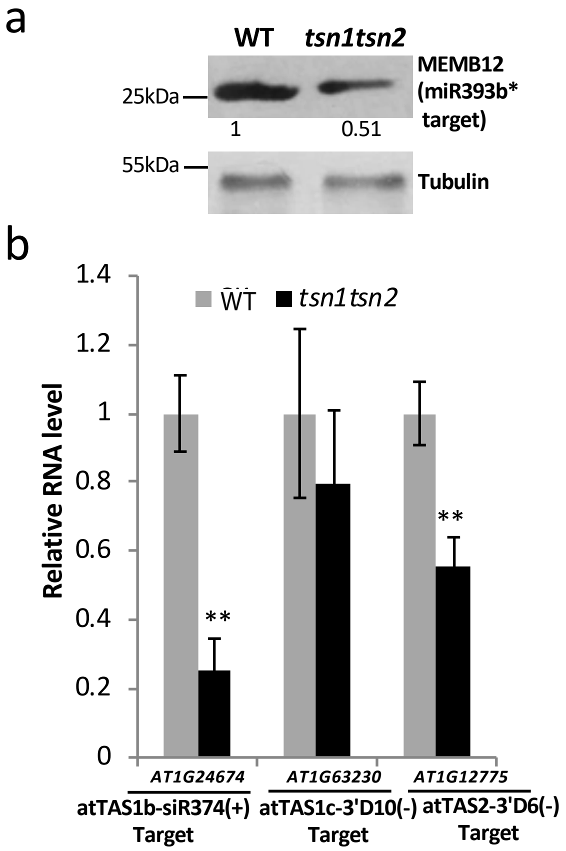

Supplementary figure 12. Expression of the AGO2-associated sRNA targets was measured by Western blot (a) or quantitative RT-PCR (b) in wild type (WT) and the *tsn1tsn2* double mutant. Error bars represent standard deviation. Asterisks indicate mean values that differ significantly from wild type (**P-valve<0.01).

Supplementary figure 13

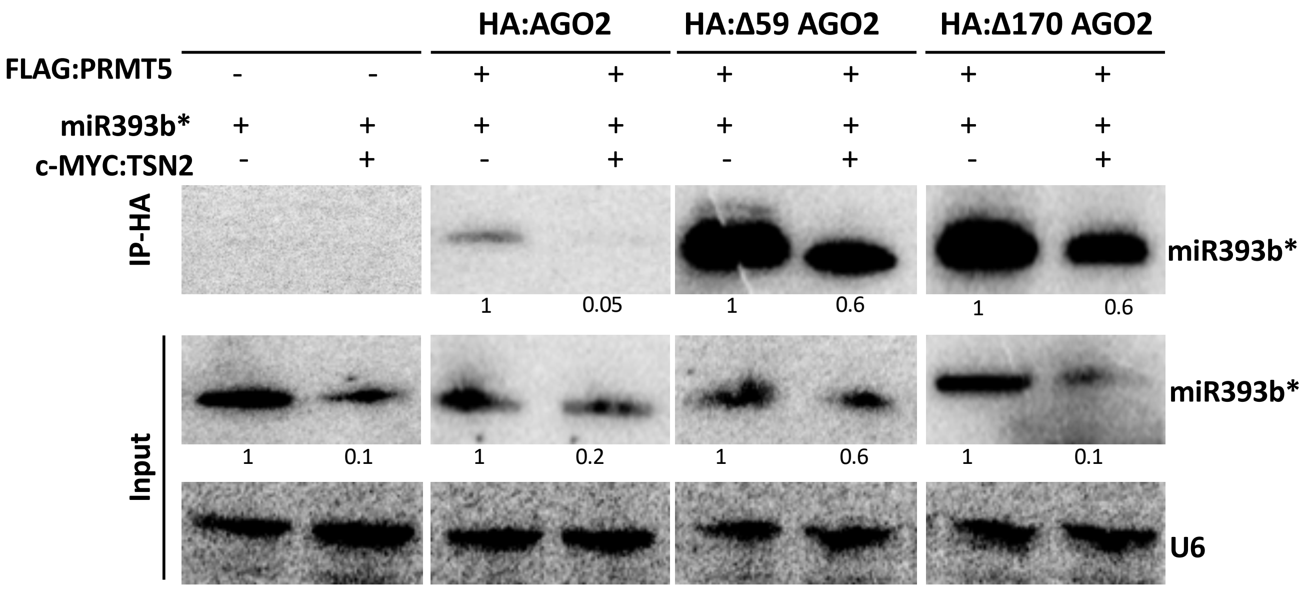

Supplementary figure 13. Deletion of the AGO2 N-terminal region prevents TSN-mediated sRNA degradation. miR393b* and different AGO2 versions (HA:AGO2; HA:Δ59 AGO2 and HA:Δ170 AGO2) were co-infiltrated with or without FLAG:PRMT5 and c:MYC:TSN2 in *N. benthamiana.* MiR393b* level was measured by Northern blot analysis. U6 was used as an internal control.

Supplementary Table 1 primers list

Constructs Primer Pairs

HA-AGO2-pENTR F	CACC ATGTATCCTTATGATGTACCTGATTATGCCTACCCATACGACGTTCCA-GACTACGCTTACCCATACGACGTTCCAGACTACGCTATGGAGAGAGGTGGTTAT
HA-AGO2-pENTR R	TCAGACGAAGAACATAAC
FLAG-PRMT5 F	ATG GACTACAAGGACGACGATGACAAG CCGCTCG GAGAGAGAGG
FLAG-PRMT5 R	GG ACTAG CTAAAGGCCAACCCAGTAC
pENTR-HSP90 F	CAC CAT GGC GGATGT TCA GAT GGCTGA TGC AGA G
pENTR-HSP90 R	TTAGTCGACTTCCTCCATCTTGC
pSAT1-nVenus-AGO2 F	CTTGAGCTCCTATGGAGAGAGGTGGTTAT
pSAT1-nVenus-AGO2 R	CTAGTCGACTCAGCAGTAGAACATGAC
pSAT4-c-CFP-PRMT5 F	CTTGAGCTCCTATGCCGCTCGGAGAGAGA
pSAT4-c-CFP-PRMT5 R	CTAGTCGACCTAAAGGCCAACCCAGTA
pSAT4-c-CFP-HSP90 F	GAC CTGCAG TC ATGGCGGACGCTGAAACC
pSAT4-c-CFP-HSP90 R	CTT GGTACC TTAGTCGACTTCCTCCAT
HA-Δ 59-pENTR F	CACC ATGTATCCTTATGATGTACCTGATTATGCCTACCCATACGACGTTCCAGACT-ACGCTTACCCATACGACGTTCCAGACTACGCT GAACAACAGGATTTTCGAAGCCAGAGTCAGC
HA-Δ 59-pENTR R	TCAGACGAAGAACATAAC
pro-AGO2 F	CAGACCTGCAGGGATTCCGGGAGAGAG
pro-AGO2 R	CAGAGGTACCTGGATCTGATCGGG
pENTR-AGO2 F	CACCATGGAGAGAGGTGGTTAT
pENTR-AGO2 R	TCAGACGAAGAACATAAC
pENTR-AGO2Δ170 F	CACCATGGTGGCTGTGC GGCGTGTTAA
pENTR-AGO2Δ170 R	TCAGACGAAGAACATAAC
pENTR-AGO2N170 F	CACCATGGAGAGAGGTGGTTAT
pENTR-AGO2N170 R	AACTCCGCCTCTATCAGGTC
pET28-PRMT5 F	ACGCGTCGACATGCCGCTCG GAGAGAGAGG
pET28-PRMT5 R	CCGCTCGAGCTAAAGGCCAACCCAGTAC

pET28-TSN1 F	ACGCGTCGACGCAACGGGGGCTGAGAAC
pET28-TSN1 R	CCGCTCGAGTTACCGGCGACCACCAGCA
pET28-TSN2 F	GACGGATCCGCGACTGGGGCAGCAACTG
pET28-TSN2 R	ATAAGAATGCGGCCGCTTA CCC GCG ACC CGG TTT CCT GAC
pENTR-TSN1 F	CAC CAT GGC AAC GGG GGC TGA GAA C
pENTR-TSN1 R	TTACCGGCGACCACCAGCA
pENTR-TSN2 F	CACCATGGCGACTGGGGCAGCAACTGAGAAC
pENTR-TSN2 R	TTA CCC GCG ACC CGG TTT CCT GAC TGG AAC ATT G
pENTR-PRMT5 F	CACCATGCCGCTCG GAGAGAGAGG
pENTR-PRMT5 R	CTAAAGGCCAACCCAGTAC
**Real-time Primer Pairs**	
PRMT5 F	CTT GCG CAC TTT GAA ACT GCT TAT G
PRMT5 R	GTA TCA GGG TGA ACC TCC ACA G
AT1G24674 F	AATAGCAAGAGCCCATCCAA
AT1G24674 R	GTTAACCCGCCACTTCGTC
AT1G63230 F	TGGAGGATCTGCAGAAGAGTG
AT1G63230 R	AAATCGATCGAGCATGAAGC
AT1G12775 F	AAGGAGAACCGCCTAGAGGA
AT1G12775 R	CGCATCCCTTGCTAATCATC
**Probe**	
TAS1b-si374	TGAATGACTCATTCGCTTGTT
atTAS1c-3'D10(-)	CAGTCGCTAATACAGTTACTT
atTAS2-3'D6(-)	CAGATGGTAGAAATGGGATAT
micro393b*	AATCCAAAGAGATCGCATGAT
micro393	GGATCAATGCGATCCCTTTGGA
