# Supplementary material for: Dual regulation of Arabidopsis AGO2 by arginine methylation
Source: Nat Commun. 2019 Feb 19;10:844. doi: 10.1038/s41467-019-08787-w (PMC6381116; doi:10.1038/s41467-019-08787-w)
Supplement: Supplementary file 2 — Description of Additional Supplementary Files [file 41467_2019_8787_MOESM2_ESM.docx]

**Description of Additional Supplementary Files**

File Name: Supplementary Data 1

Description: Supplementary dataset 1 is the list of AGO2-associated proteins identified from the LC-MS/MS analysis. These proteins are associated only with pAGO2::3HA:AGO2 but not with pAGO7::gus by using the label free quantification (LFQ) intensity value. The complete mass spectrometry dataset was uploaded in PeptideAtlas. The Accession codes is PASS01211(http://www.peptideatlas.org/PASS/PASS01211).
