# Supplementary material for: Dual regulation of Arabidopsis AGO2 by arginine methylation
Source: Nat Commun. 2019 Feb 19;10:844. doi: 10.1038/s41467-019-08787-w (PMC6381116; doi:10.1038/s41467-019-08787-w)
Supplement: Supplementary file 5 — Source Data [file 41467_2019_8787_MOESM5_ESM.pdf]

Fig 1b

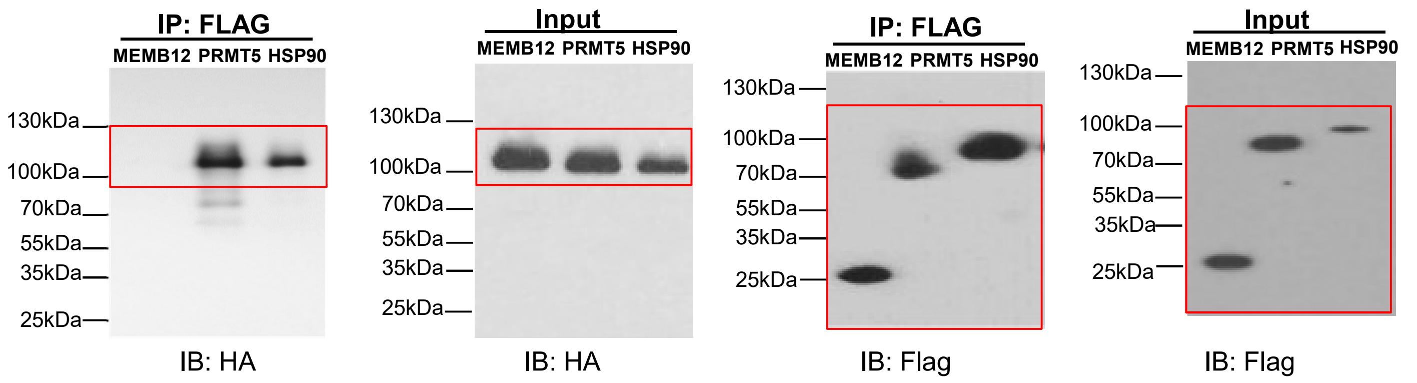

Fig 1c

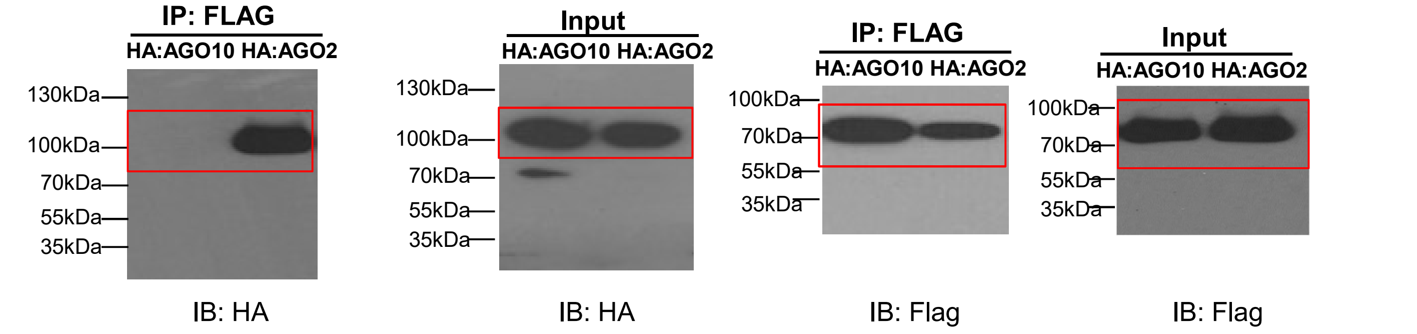

Fig 1e

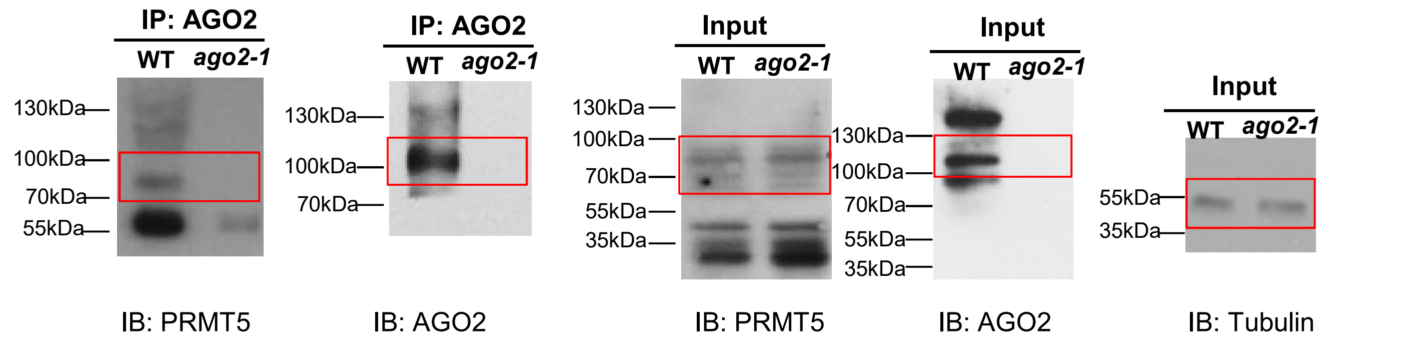

Fig 2a

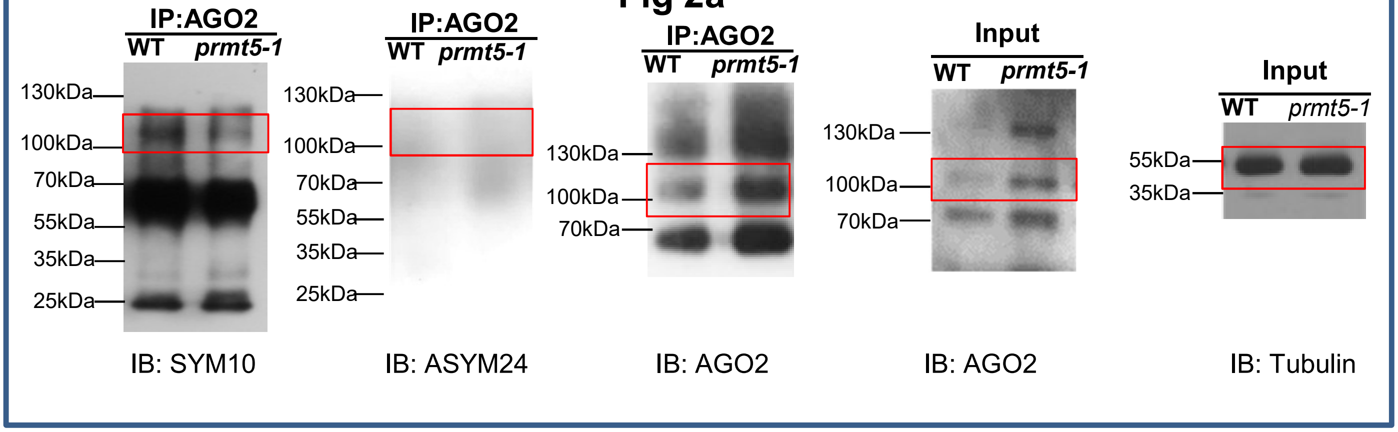

Fig 2c

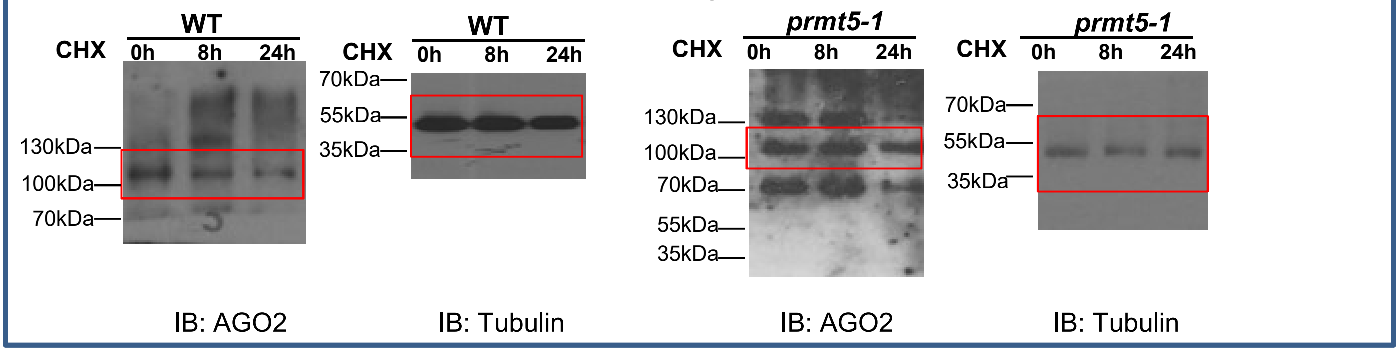

Fig 2d

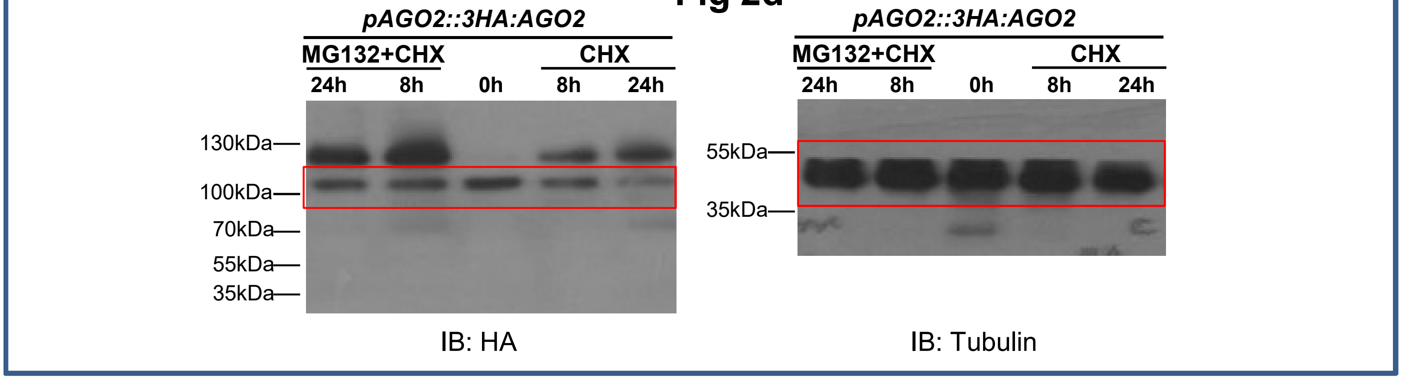

Fig 2e

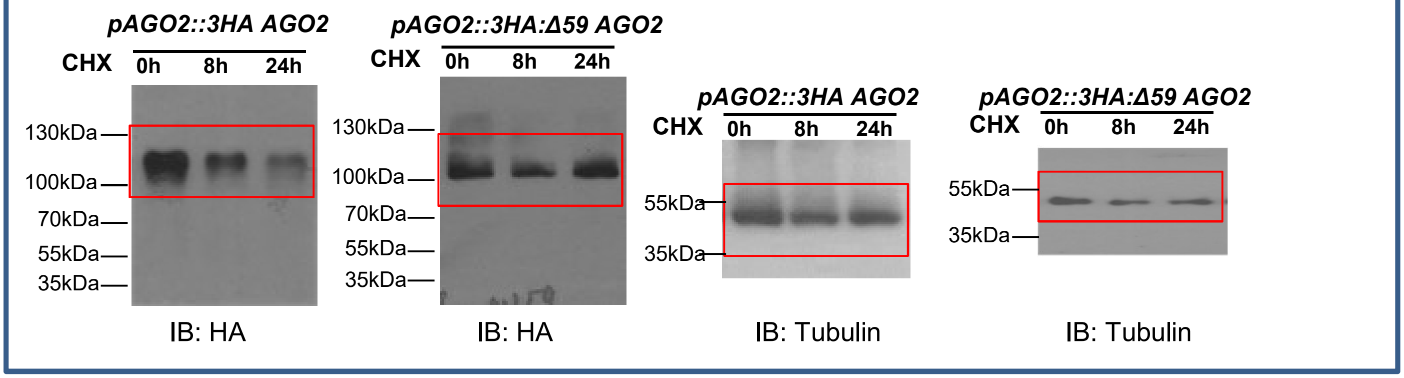

Fig 3b

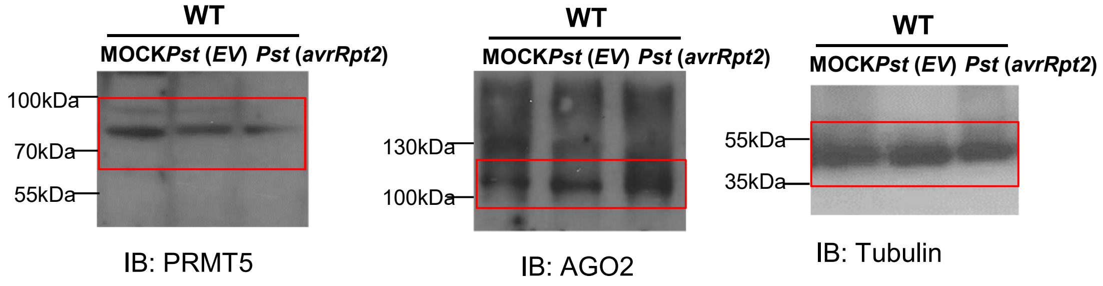

Fig 3c

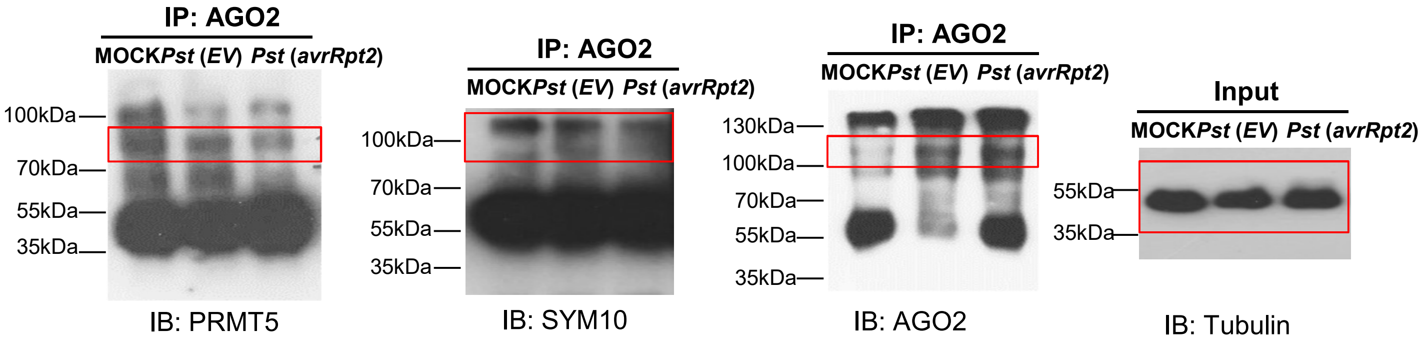

Fig 3d

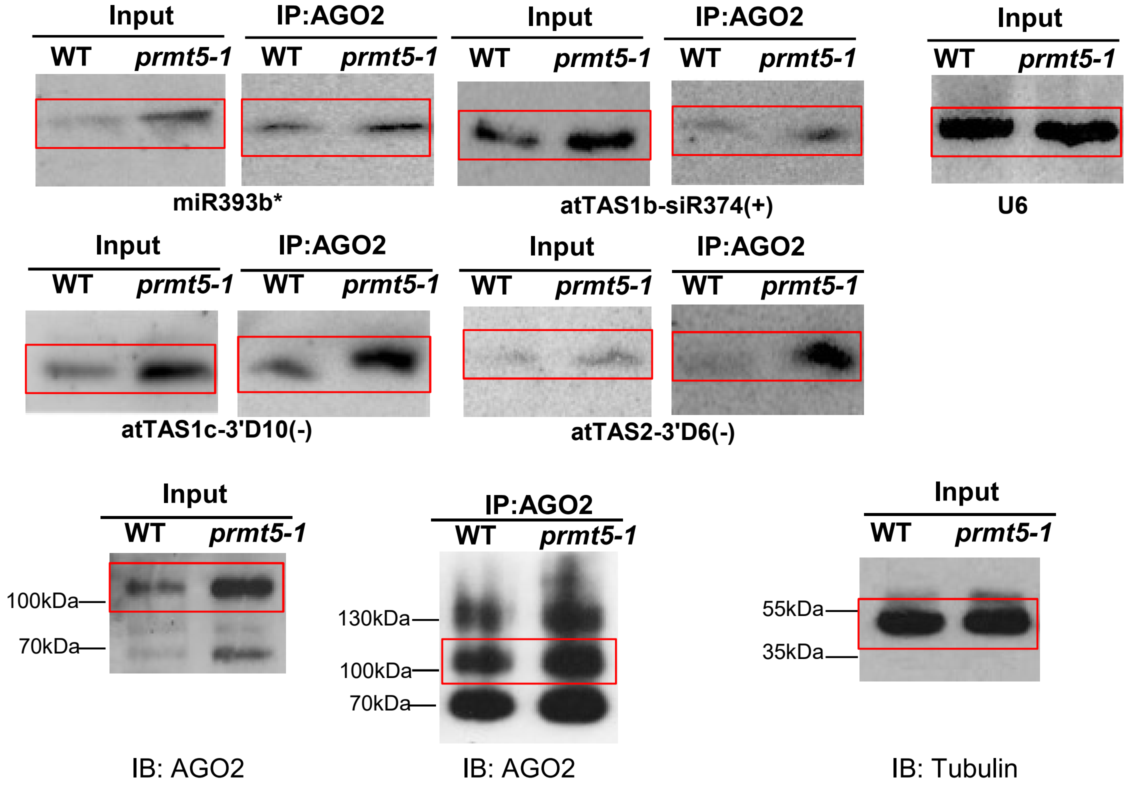

Fig 3f

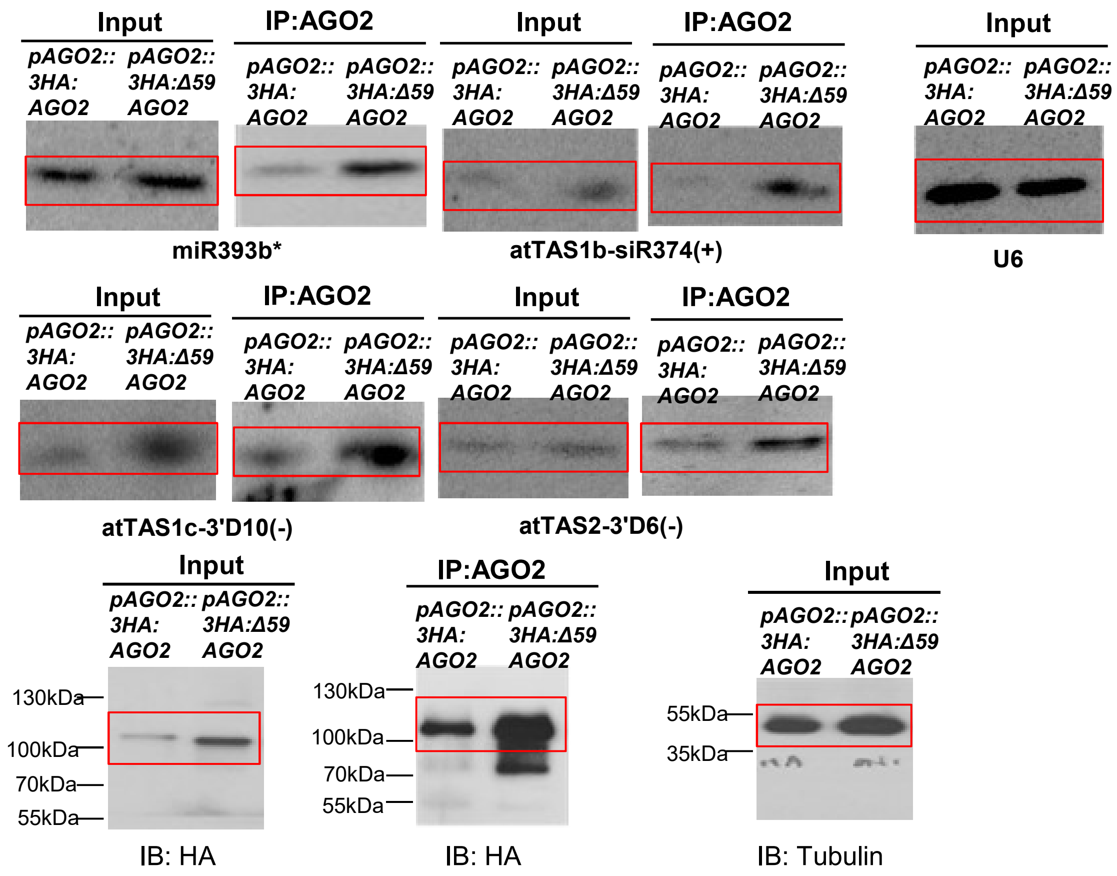
Input
IP:AGO2
pAGO2:: 3HA: AGO2
pAGO2:: 3HA:Δ59 AGO2
miR393b*
atTAS1b-siR374(+)
U6
atTAS1c-3'D10(-)
atTAS2-3'D6(-)
130kDa
100kDa
70kDa
55kDa
35kDa
IB: HA
IB: HA
IB: Tubulin

Fig 4a

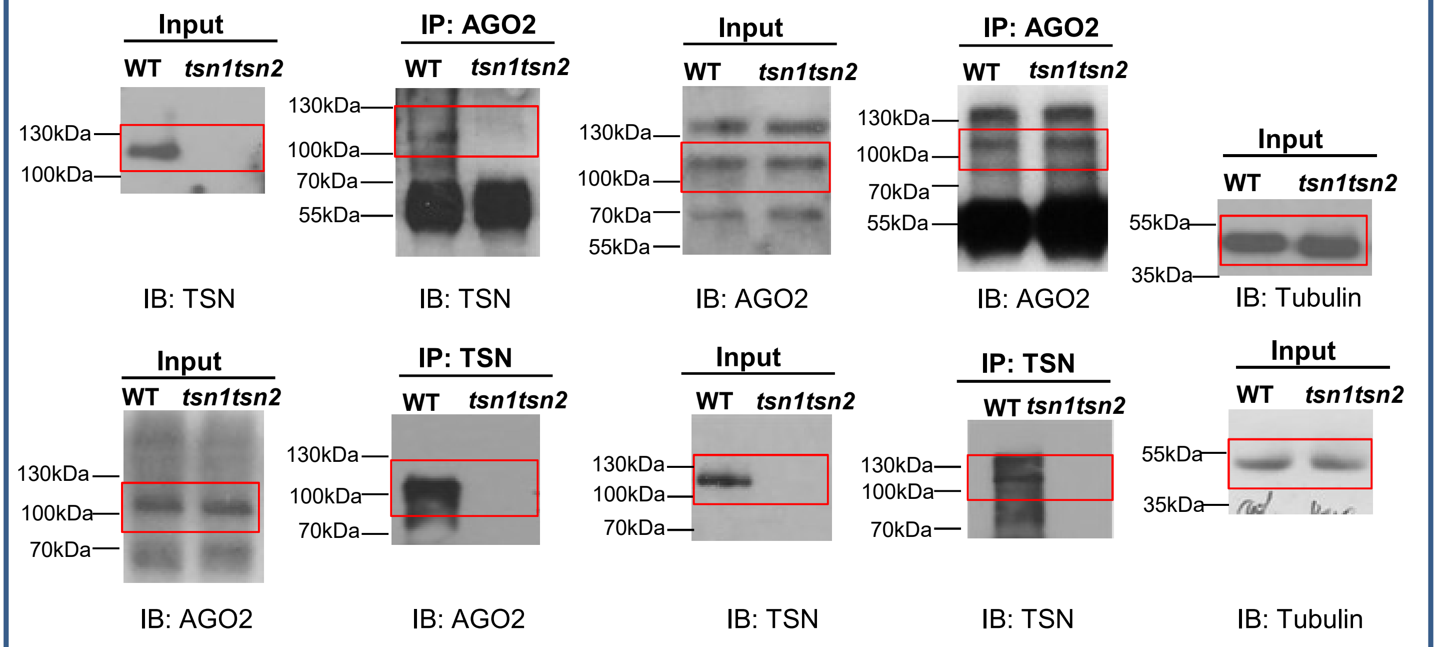
Input
WT tsn1tsn2
130kDa
100kDa
IB: TSN
IP: AGO2
WT tsn1tsn2
130kDa
100kDa
70kDa
55kDa
IB: TSN
Input
WT tsn1tsn2
130kDa
100kDa
70kDa
55kDa
IB: AGO2
IP: AGO2
WT tsn1tsn2
130kDa
100kDa
70kDa
55kDa
IB: AGO2
Input
WT tsn1tsn2
55kDa
35kDa
IB: Tubulin
Input
WT tsn1tsn2
130kDa
100kDa
70kDa
IB: AGO2
IP: TSN
WT tsn1tsn2
130kDa
100kDa
70kDa
IB: AGO2
Input
WT tsn1tsn2
130kDa
100kDa
70kDa
IB: TSN
IP: TSN
WT tsn1tsn2
130kDa
100kDa
70kDa
IB: TSN
Input
WT tsn1tsn2
55kDa
35kDa
IB: Tubulin

Fig 4b

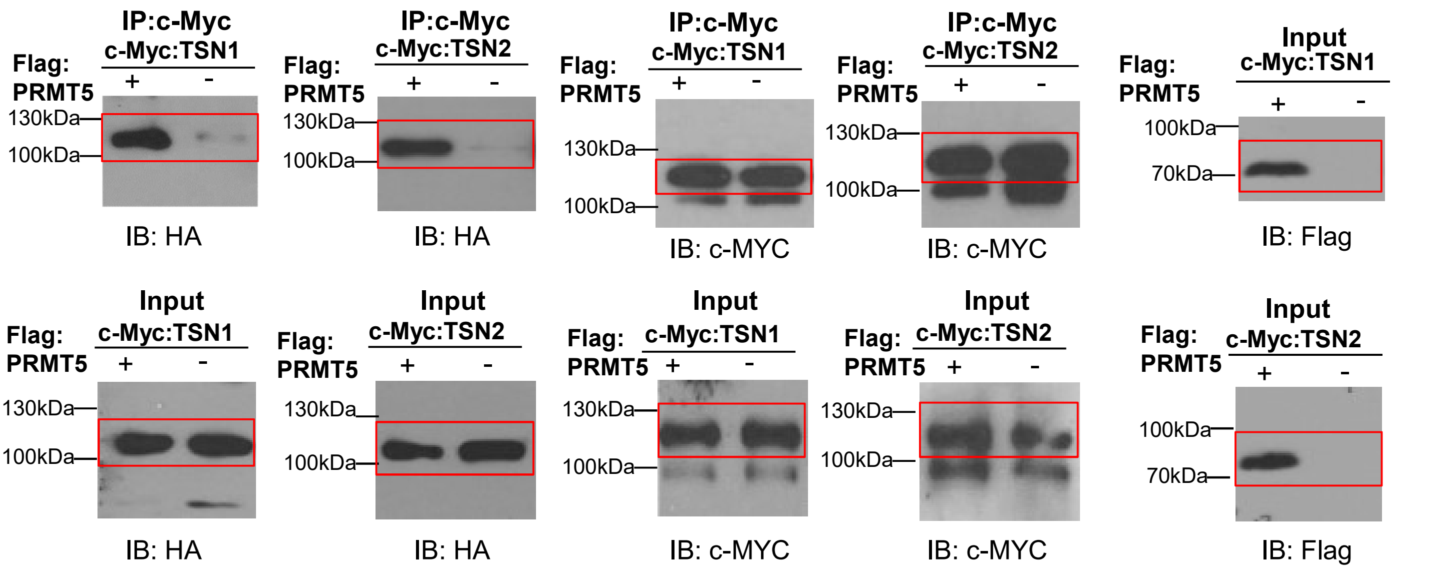
IP:c-Myc
c-Myc:TSN1
Flag: PRMT5 + -
130kDa
100kDa
IB: HA
IP:c-Myc
c-Myc:TSN2
Flag: PRMT5 + -
130kDa
100kDa
IB: HA
IP:c-Myc
c-Myc:TSN1
Flag: PRMT5 + -
130kDa
100kDa
IB: c-MYC
IP:c-Myc
c-Myc:TSN2
Flag: PRMT5 + -
130kDa
100kDa
IB: c-MYC
Input
c-Myc:TSN1
Flag: PRMT5 + -
100kDa
70kDa
IB: Flag
Input
Flag: c-Myc:TSN1
PRMT5 + -
130kDa
100kDa
IB: HA
Input
Flag: c-Myc:TSN2
PRMT5 + -
130kDa
100kDa
IB: HA
Input
Flag: c-Myc:TSN1
PRMT5 + -
130kDa
100kDa
IB: c-MYC
Input
Flag: c-Myc:TSN2
PRMT5 + -
130kDa
100kDa
IB: c-MYC
Input
Flag: c-Myc:TSN2
PRMT5 + -
100kDa
70kDa
IB: Flag

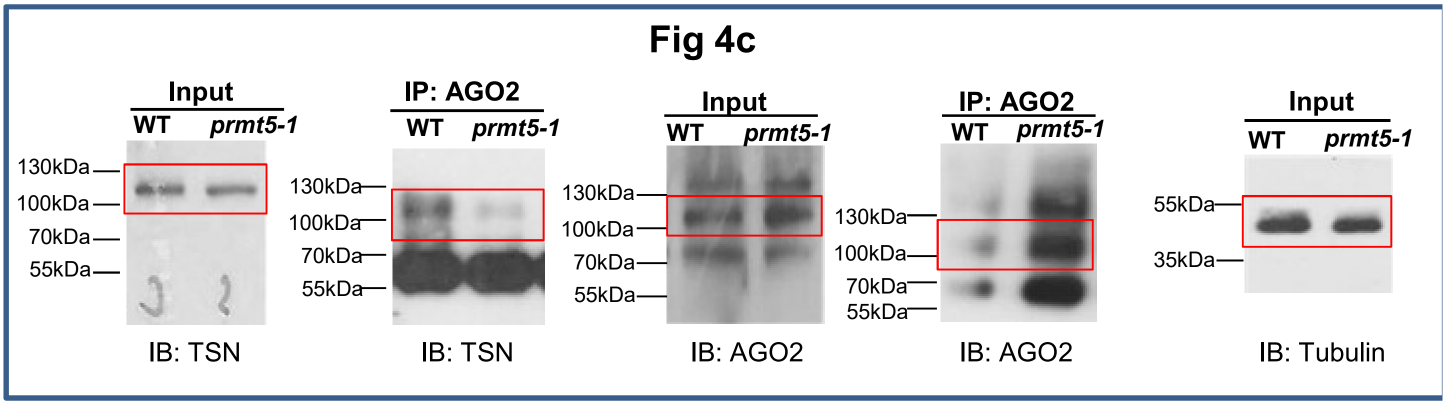
Fig 4c
Input
WT prmt5-1
130kDa
100kDa
70kDa
55kDa
IB: TSN
IP: AGO2
WT prmt5-1
130kDa
100kDa
70kDa
55kDa
IB: TSN
Input
WT prmt5-1
130kDa
100kDa
70kDa
55kDa
IB: AGO2
IP: AGO2
WT prmt5-1
130kDa
100kDa
70kDa
55kDa
IB: AGO2
Input
WT prmt5-1
55kDa
35kDa
IB: Tubulin

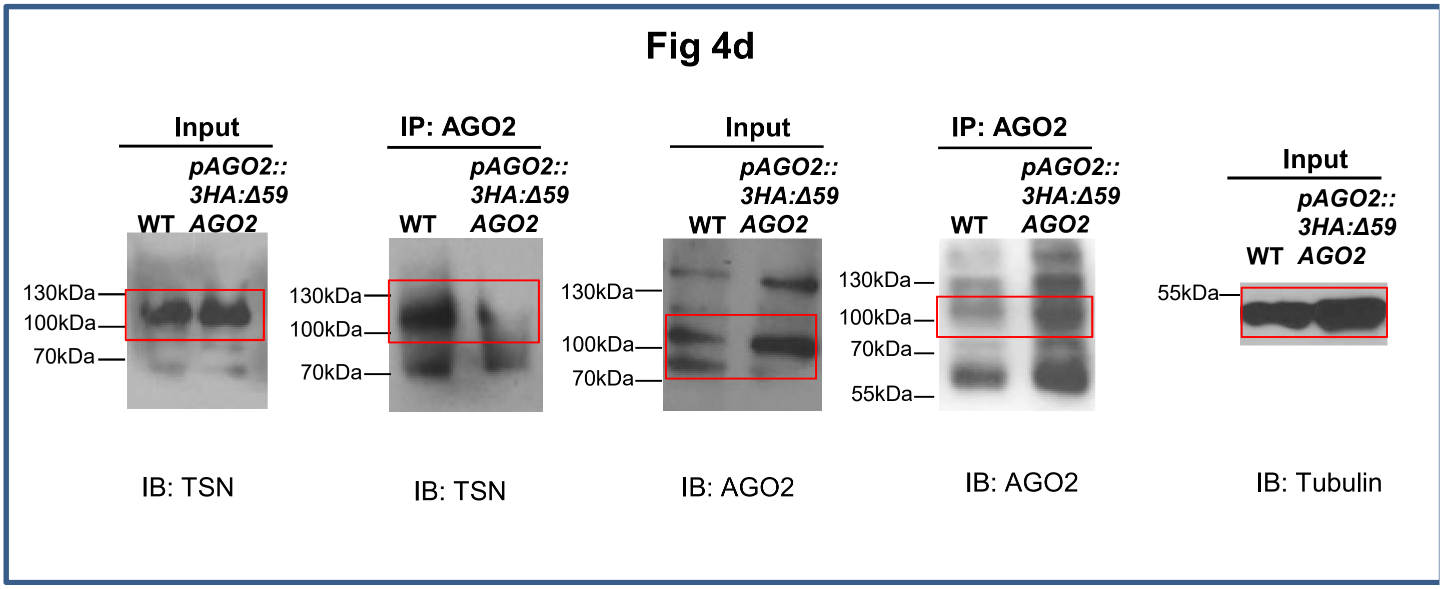
Fig 4d
Input
WT pAGO2::3HA:Δ59 AGO2
130kDa
100kDa
70kDa
IB: TSN
IP: AGO2
WT pAGO2::3HA:Δ59 AGO2
130kDa
100kDa
70kDa
IB: TSN
Input
WT pAGO2::3HA:Δ59 AGO2
130kDa
100kDa
70kDa
IB: AGO2
IP: AGO2
WT pAGO2::3HA:Δ59 AGO2
130kDa
100kDa
70kDa
55kDa
IB: AGO2
Input
WT pAGO2::3HA:Δ59 AGO2
55kDa
IB: Tubulin

Fig 5a

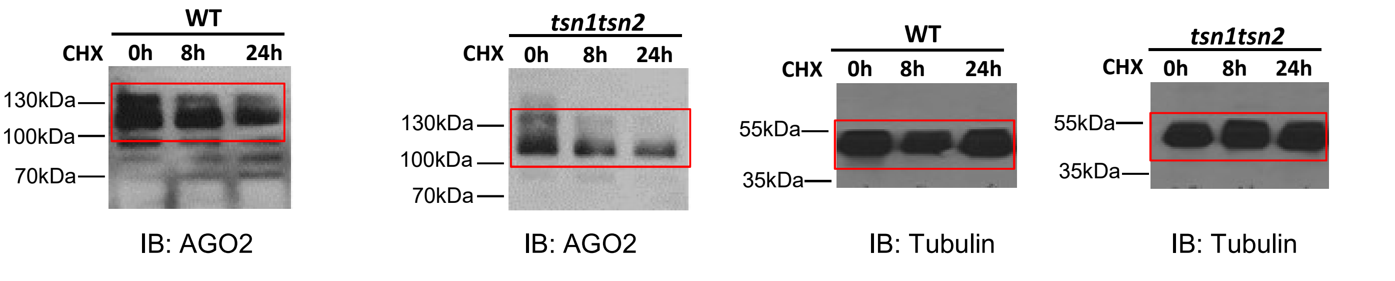
WT
CHX 0h 8h 24h
130kDa
100kDa
70kDa
IB: AGO2
tsn1tsn2
CHX 0h 8h 24h
130kDa
100kDa
70kDa
IB: AGO2
WT
CHX 0h 8h 24h
55kDa
35kDa
IB: Tubulin
tsn1tsn2
CHX 0h 8h 24h
55kDa
35kDa
IB: Tubulin

Fig 5b

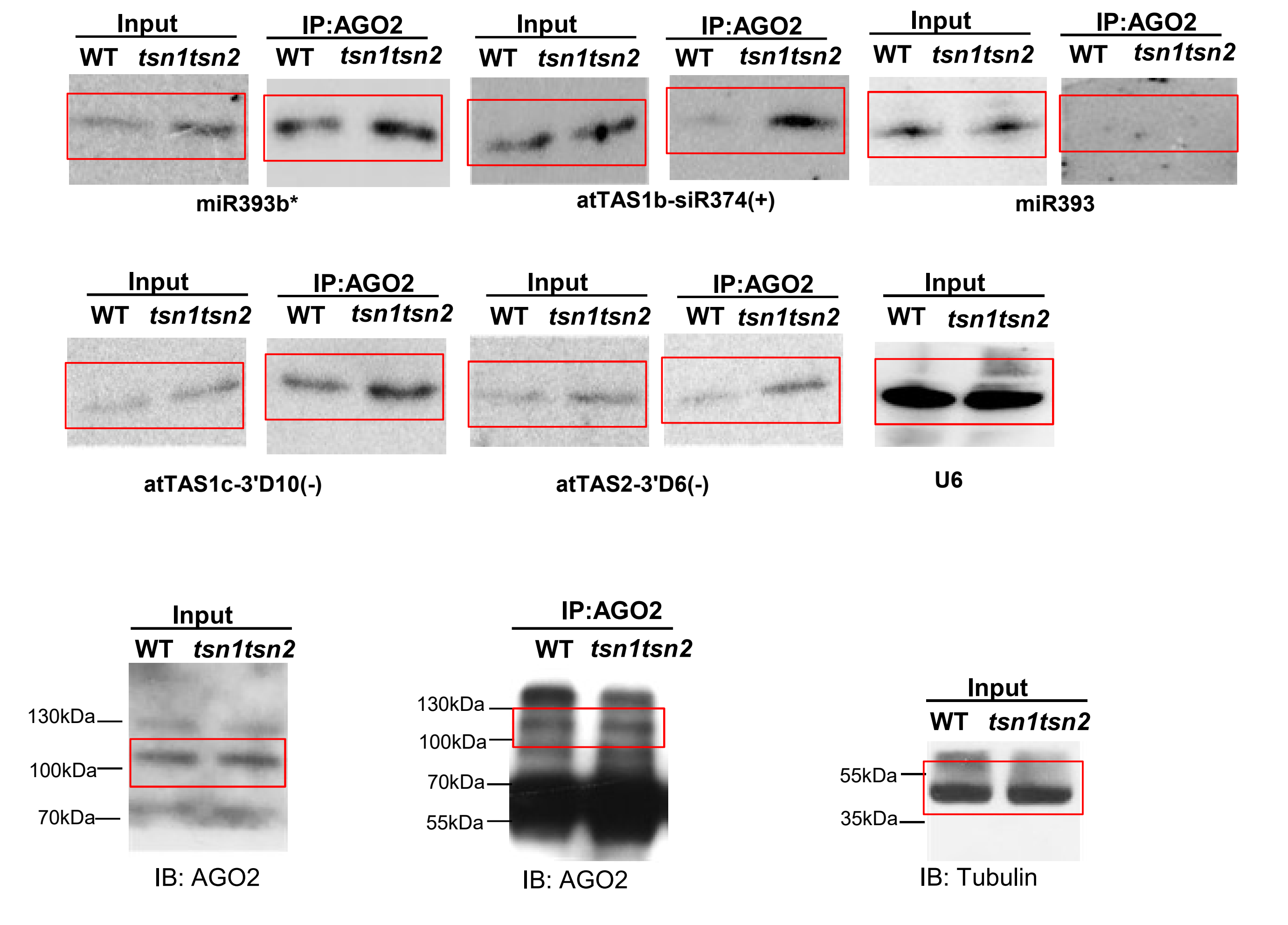
Input
WT tsn1tsn2
IP:AGO2
WT tsn1tsn2
miR393b*
Input
WT tsn1tsn2
IP:AGO2
WT tsn1tsn2
atTAS1b-siR374(+)
Input
WT tsn1tsn2
IP:AGO2
WT tsn1tsn2
miR393
Input
WT tsn1tsn2
IP:AGO2
WT tsn1tsn2
atTAS1c-3'D10(-)
Input
WT tsn1tsn2
IP:AGO2
WT tsn1tsn2
atTAS2-3'D6(-)
Input
WT tsn1tsn2
U6
Input
WT tsn1tsn2
130kDa
100kDa
70kDa
IB: AGO2
IP:AGO2
WT tsn1tsn2
130kDa
100kDa
70kDa
55kDa
IB: AGO2
Input
WT tsn1tsn2
55kDa
35kDa
IB: Tubulin

Fig 5c

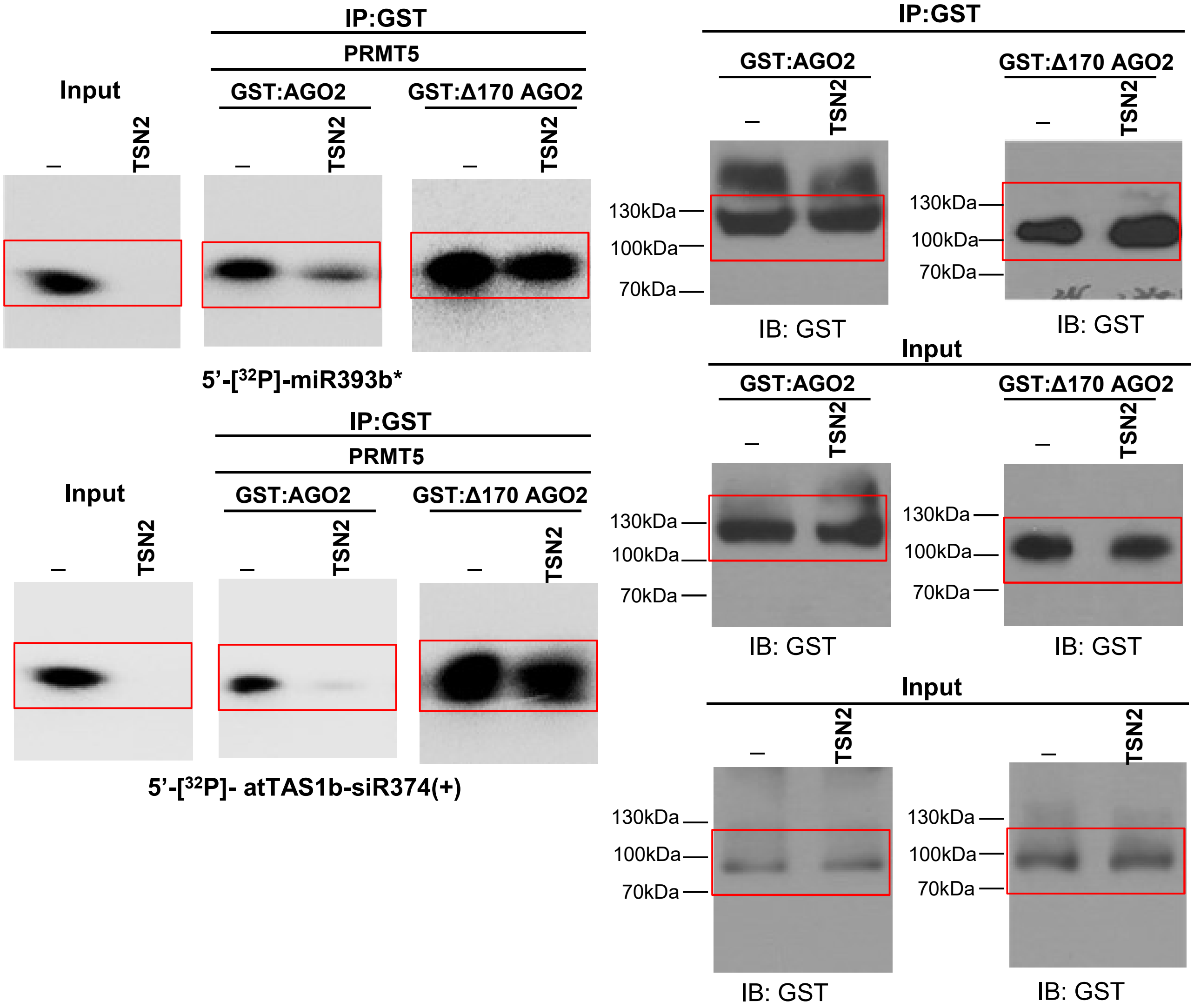
IP:GST
PRMT5
Input
GST:AGO2
GST:Δ170 AGO2
–
TSN2
5'-[32P]-miR393b*
5'-[32P]- atTAS1b-siR374(+)
130kDa
100kDa
70kDa
IB: GST

Supplementary Figure 1b

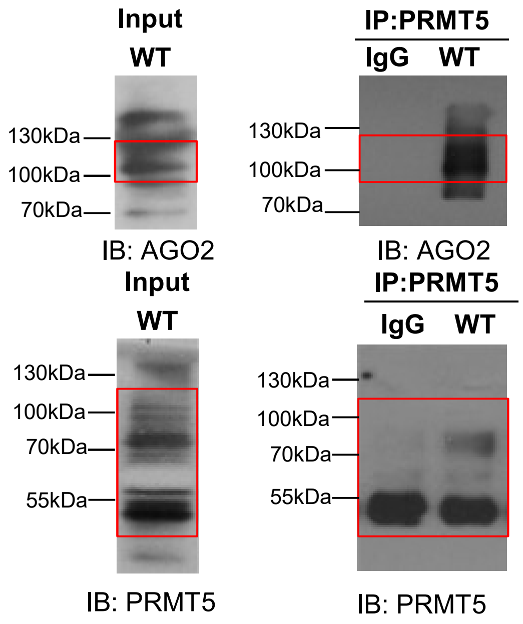

Supplementary Figure 3b

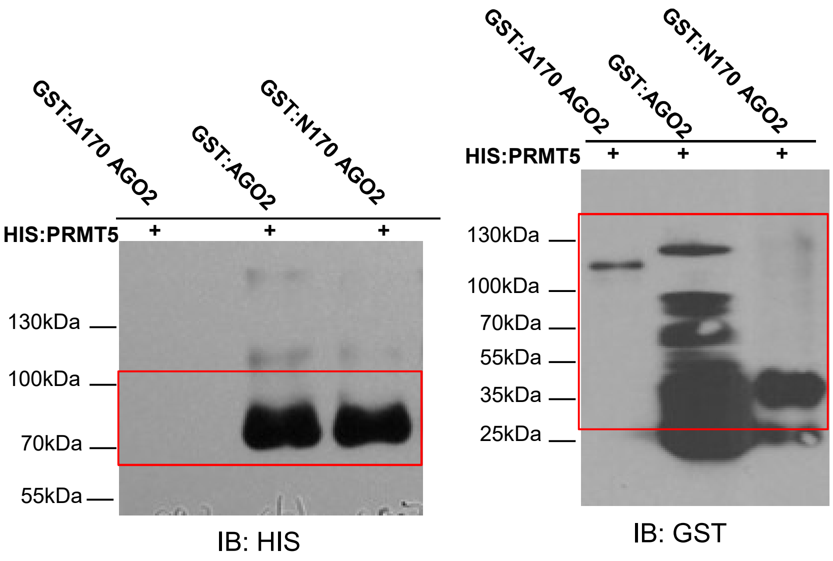

Supplementary Figure 5

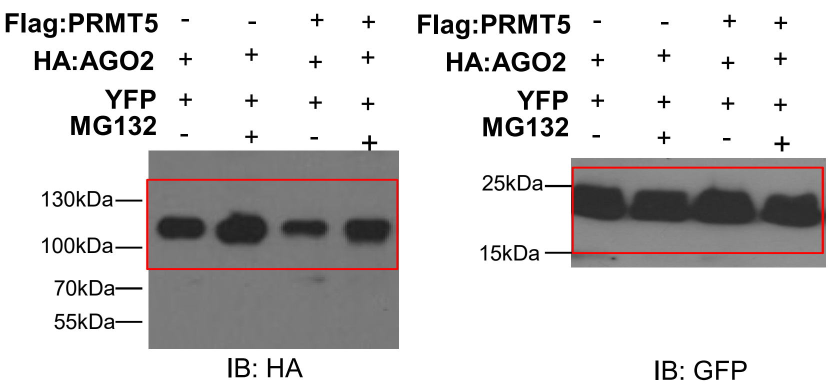

Supplementary Figure 6a

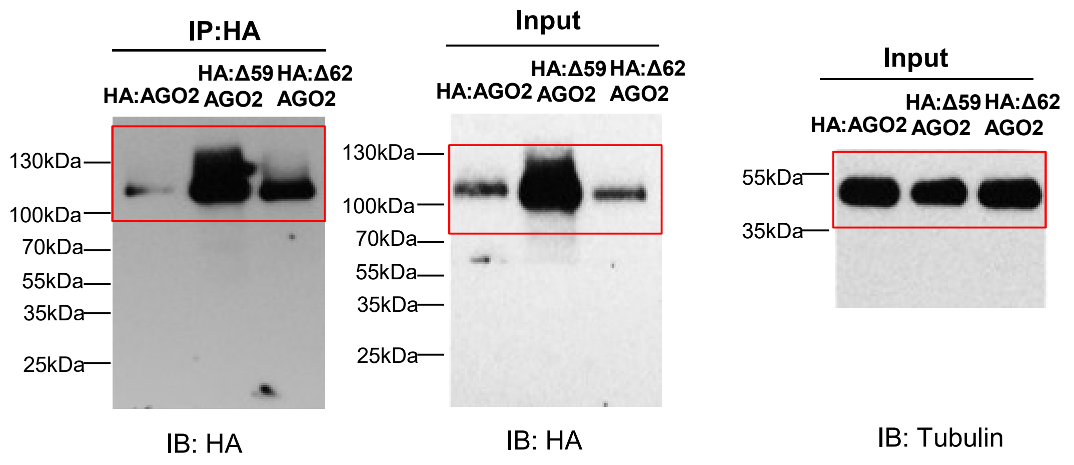

Supplementary Figure 7

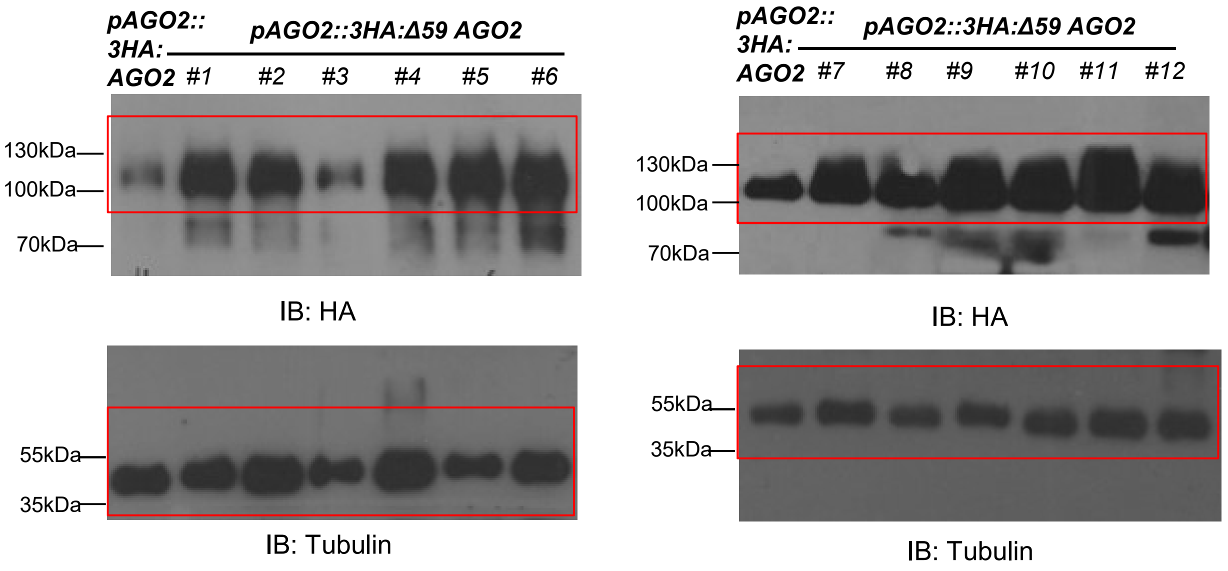

Supplementary Figure 8a

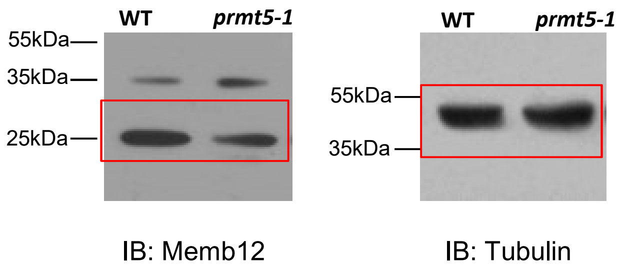

Supplementary Figure 9a

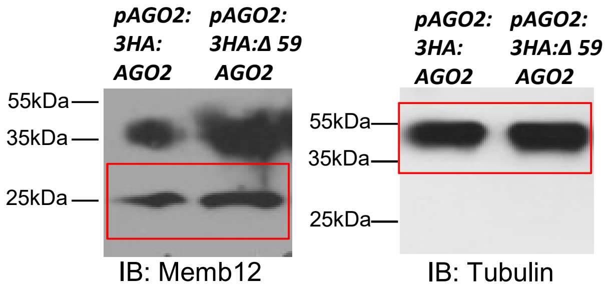

Supplementary Figure 11a

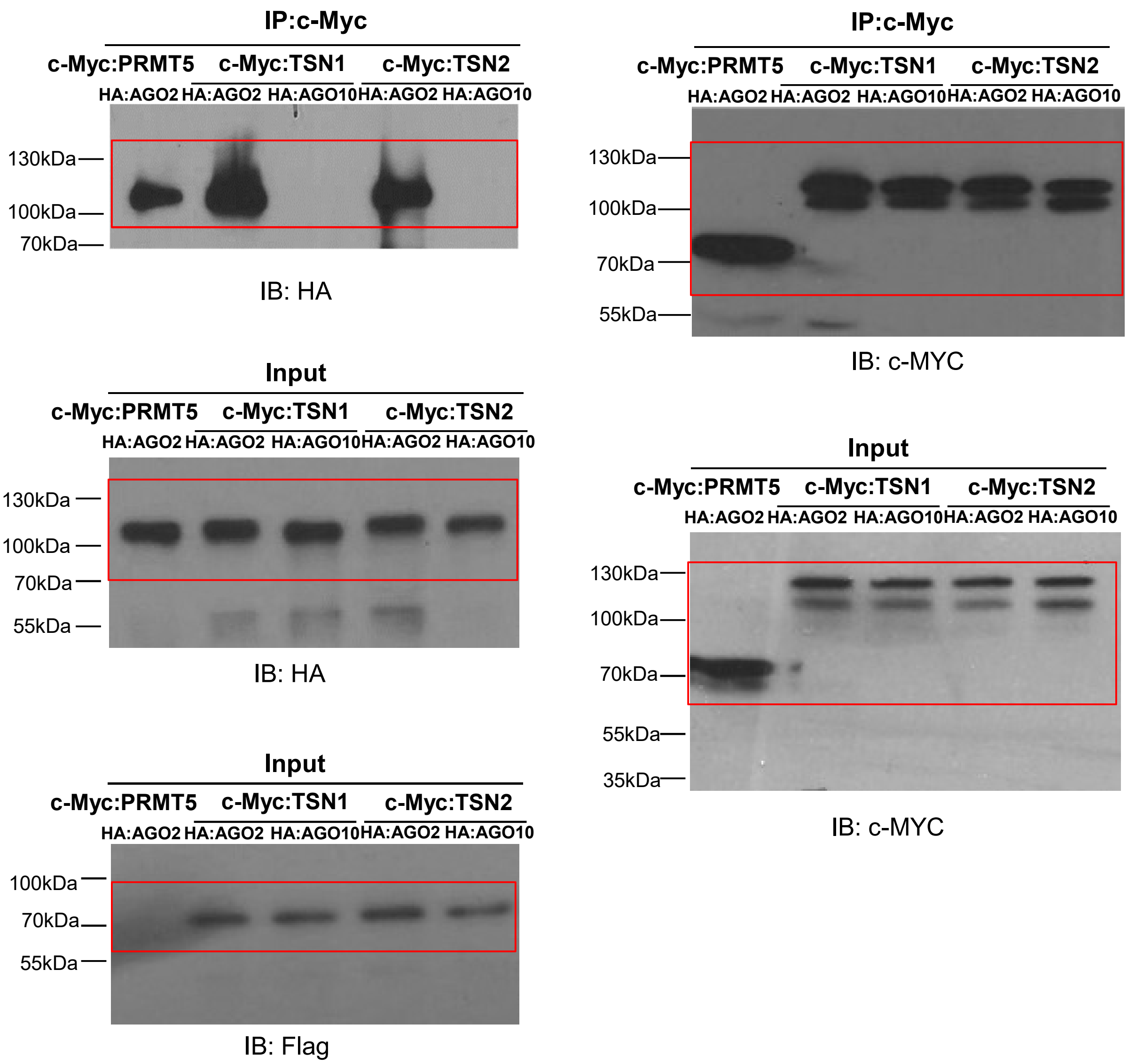

Supplementary Figure 11b

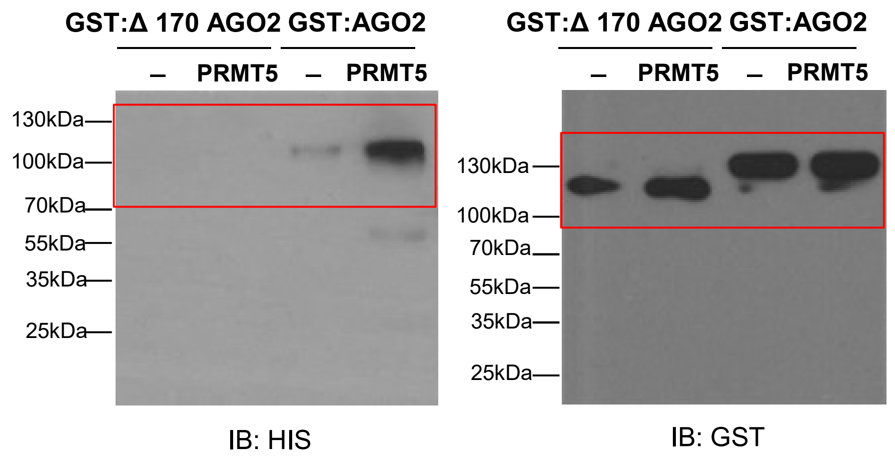

Supplementary Figure 11c

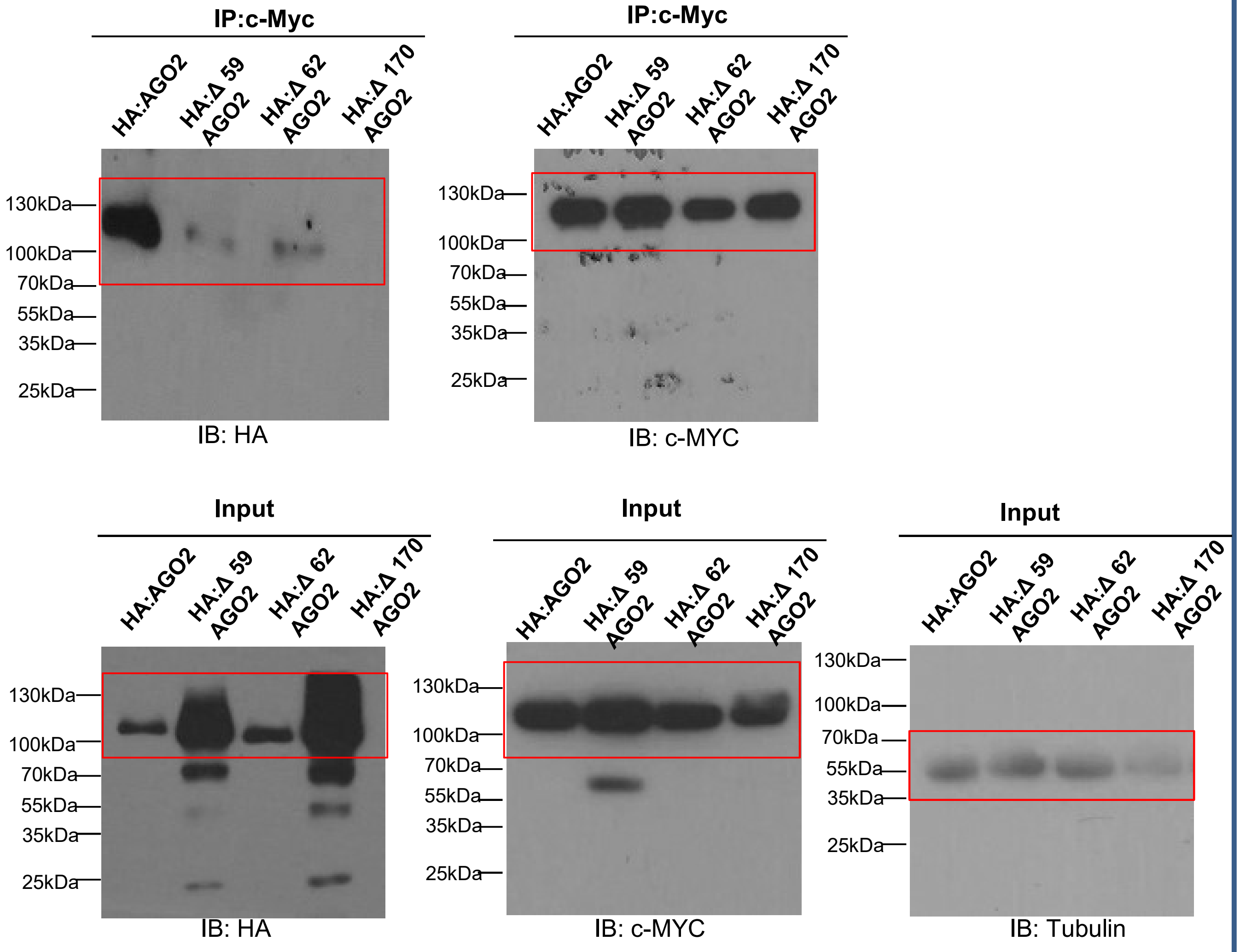

Supplementary Figure 12a

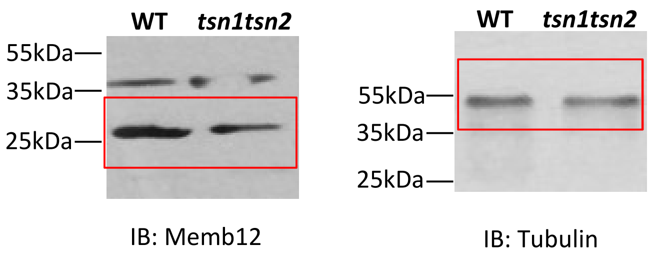

Supplementary Figure 13

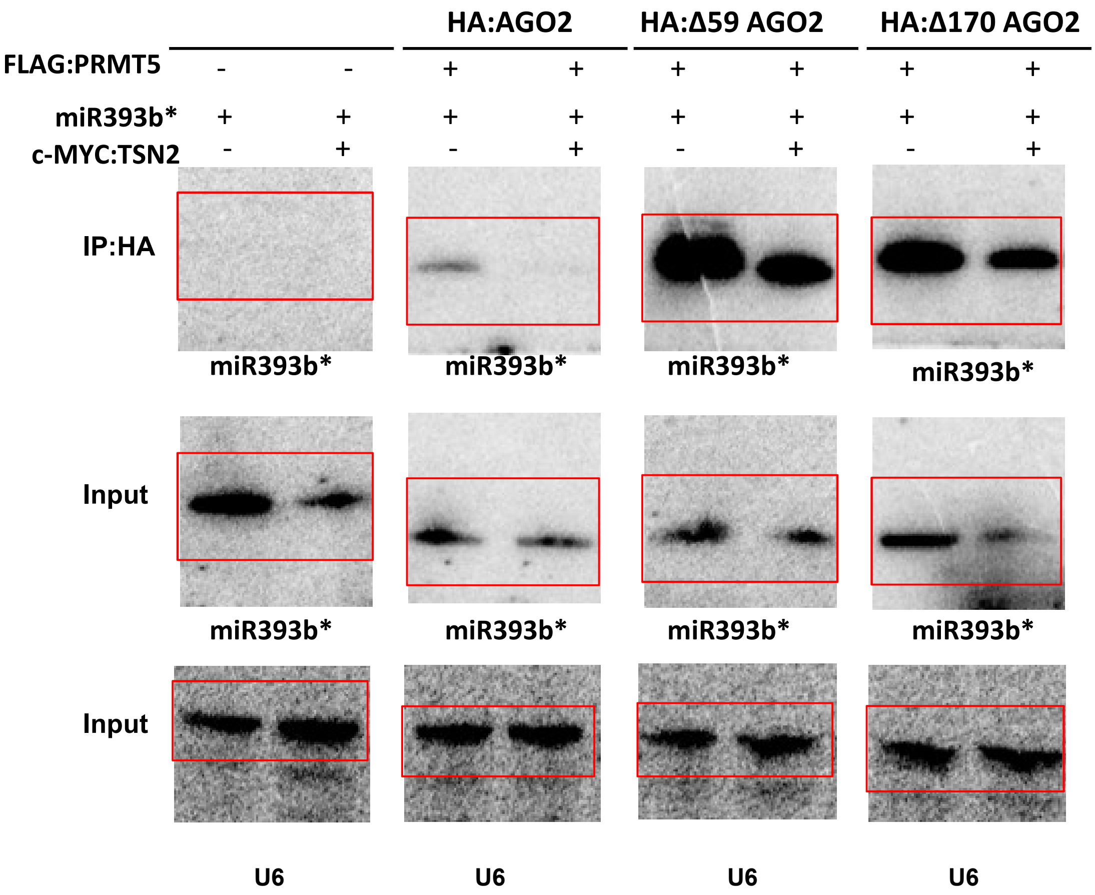

Figure 3e

Pst (EV)0d	*Ago2-1*	*WT*	*prmt5-1*
Log CFU/cm^2	2.913814	2.832509	2.732394
	2.832509	2.792392	2.69897
	2.857332	2.748188	2.80618
AVE	2.867885	2.79103	2.745848
SD	0.041667	0.042177	0.054857

Pst (EV)3d	*Ago2-1*	*WT*	*prmt5-1*
	6.528917	6.113943	6.100371
	6.565848	6.342423	5.973128
	6.810233	6.181844	5.954243
	6.623249	6.485721	6.158362
	6.802089	6.40824	6.127105
	6.724276	6.230449	6.064458
	6.675778	6.338456	5.954243
	6.705864	6.240549	5.991226
			5.963788
			6
	6.679532	6.292703	6.028692
	0.102532	0.123063	0.081097

Pst (avrRpt2)0d	*Ago2-1*	*WT*	*prmt5-1*
	2.80618	2.778151	2.716003
	2.857332	2.716003	2.643453
	2.924279	2.748188	2.869232
AVE	2.862597	2.747448	2.742896
SD	0.059225	0.031081	0.115267

Pst (avrRpt2)3d	*Ago2-1*	*WT*	*prmt5-1*
	5.923244	5.292256	4.819544
	5.920123	5.38739	4.732394
	5.929419	5.421604	4.579784
	5.876218	5.113943	4.880814
	5.885361	5.394452	4.913814
	5.980458	5.255273	4.880814
	5.954243	5.313867	4.982271
	5.970347	5.240549	4.892095
	5.85248	5.193125	4.623249
		5.209515	
	5.921321	5.282197	4.811642
	0.043477	0.09899	0.137472

Figure 3g

Pst (EV)0d	*ago2-1*	*pAGO2:: 3HA: AGO2*	*pAGO2:: 3HA:Δ59 AGO2-1*	*pAGO2:: 3HA:Δ59 AGO2-2*	*Pst (EV)3d*	*ago2-1*	*pAGO2:: 3HA: AGO2*	*pAGO2:: 3HA:Δ59 AGO2-1*	*pAGO2:: 3HA:Δ59 AGO2-2*
**Log CFU/cm²**	2.856124	2.937518	2.893207	2.91698		6.079181	5.380211	6.283301	5.832509
	2.875061	2.826075	2.875061	2.802089		5.944483	5.380211	6	6.093422
	2.842609	2.840106	2.842609	2.790988		6.033424	5.255273	6.146128	5.380211
	2.857332	2.959995	2.772322	2.695482		5.623249	5.20412	6.531479	6.414973
								5.30103	6.447158
								5.880814	6.305351
								6.049218	5.973128
								6.120574	6.100371
								5.20412	5.857332
								5.732394	6
								5.857332	
AVE	2.857782	2.890923	2.8458	2.801385		5.920084	5.304954	5.918763	6.040446
SD	0.013313	0.06765	0.053269	0.090713		0.20564	0.089374	0.394538	0.316422

Pst (avrRpt2)0d	*ago2-1*	*pAGO2:: 3HA: AGO2*	*pAGO2:: 3HA:Δ59 AGO2-1*	*pAGO2:: 3HA:Δ59 AGO2-2*	*Pst (avrRpt2)3d*	*ago2-1*	*pAGO2:: 3HA: AGO2*	*pAGO2:: 3HA:Δ59 AGO2-1*	*pAGO2:: 3HA:Δ59 AGO2-2*
	2.819544	2.60206	2.716003	2.716003		5.20412	4.556303	4.763428	4.477121
	2.792392	2.845098	2.857332	2.819544		5.033424	4.30103	4.716003	4.414973
	2.913814	2.880814	2.913814	2.748188		4.681241	4.447158	4.748188	4.623249
	2.857332	2.819544	2.880814			4.623249	4.778151	4.662758	5.056905
	2.924279		2.845098				4.832509	4.681241	4.832509
							4.579784	5.176091	4.716003
							4.579784	4.763428	4.973128
							4.255273	4.880814	5.100371
							4.146128	5.113943	5.071882
							4.447158	5.176091	4.892095
								4.869232	
								4.732394	
								4.819544	
AVE	2.861472	2.786879	2.842612	2.761245		4.885509	4.492328	4.854089	4.815824
SD	0.057514	0.125749	0.075468	0.052991		0.279223	0.219477	0.183722	0.248985

Figure 5d

Pst (EV)0d	*WT*	*tsn1tsn2*
**Log CFU/cm²**	2.50515	2.505149978
	2.477121	2.505149978
	2.477121	2.505149978
	2.477121	2.556302501
	2.477121	
AVE	2.482727	2.517938109
SD	0.012535	0.025576261

Pst (EV)3d	*WT*	*tsn1tsn2*
	6.354108	5.60206
	6.531479	5.662758
	6.127105	5.255273
	6.113943	5.079181
	6.269513	5.414973
	6.025306	5.69897
	6.322219	5.579784
	6.255273	
	6.249868	5.470428
	0.160265	0.231282

Pst (avrRpt2)0d	*WT*	*tsn1tsn2*
	2.447158	2.447158031
	2.414973	2.447158031
	2.447158	2.477121255
	2.447158	2.447158031
	2.477121	
	2.477121	
	2.451782	2.454648837
	0.023252	0.014981612

Pst (avrRpt2)3d	*WT*	*tsn1tsn2*
	4.857332	4.447158
	4.60206	4.447158
	4.832509	3.90309
	4.880814	3.60206
	5	4.30103
	4.982271	
	5.158362	
	4.901907	4.140099
	0.172726	0.374209
